# Expanding the hydride chemistry: antiperovskites A_3_MO_4_H (A = Rb, Cs; M = Mo, W) introducing the transition oxometalate hydrides†

**DOI:** 10.1039/d2sc01861f

**Published:** 2022-05-25

**Authors:** Alexander Mutschke, Annika Schulz, Marko Bertmer, Clemens Ritter, Antti J. Karttunen, Gregor Kieslich, Nathalie Kunkel

**Affiliations:** Chair of Inorganic Chemistry with Focus on Novel Materials, Technical University of Munich Lichtenbergstrasse 4 85748 Garching Germany Alex.Mutschke@tum.de ga74lud@mytum.de; Felix Bloch Institute for Solid State Physics Leipzig University Linnéstrasse 5 04103 Leipzig Germany; Institut Laue-Langevin 71 Avenue des Martyrs 38042 Grenoble Cedex 9 France; Department of Chemistry and Materials Science, Aalto University P.O. Box 16100 FI-00076 Aalto Finland; Chair of Inorganic and Metal-Organic Chemistry, Technical University of Munich Lichtenbergstrasse 4 85748 Garching Germany

## Abstract

The four compounds A_3_MO_4_H (A = Rb, Cs; M = Mo, W) are introduced as the first members of the new material class of the transition oxometalate hydrides. The compounds are accessible *via* a thermal synthesis route with carefully controlled conditions. Their crystal structures were solved by neutron diffraction of the deuterated analogues. Rb_3_MoO_4_D, Cs_3_MoO_4_D and Cs_3_WO_4_D crystallize in the antiperovskite-like K_3_SO_4_F-structure type, while Rb_3_WO_4_D adopts a different orthorhombic structure. ^2^H MAS NMR, Raman spectroscopy and elemental analysis prove the abundance of hydride ions next to oxometalate ions and experimental findings are supported by quantum chemical calculations. The tetragonal phases are direct and wide band gap semiconductors arising from hydride states, whereas Rb_3_WO_4_H shows a unique, peculiar valence band structure dominated by hydride states.

## Abstract 2

The synthesis, structures and electronic properties of the first four heteroanionic compounds containing both hydride and transition oxometalate ions are reported.

## Graphical abstract

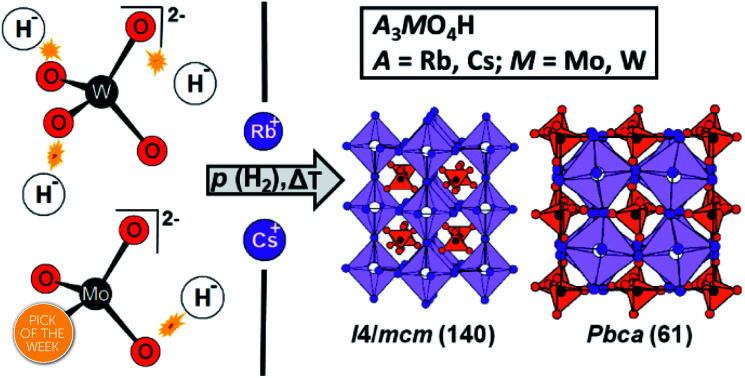

## Introduction

Mixed anionic hydrides as a subclass of mixed anionic compounds^1^ have recently raised a lot of attention due to a variety of academically interesting and technological relevant properties such as fast hydride ion conduction,^2^ tunable optical properties^3^ and superconductivity^4^ amongst others. In addition, the number of mixed anionic hydrides with acceptable air and moisture stability is steadily increasing which facilitates their application in the future.^5–8^ To date, oxy hydrides with isolated oxide ions represent the largest group of this materials class which includes a variety of transition metal based oxy hydrides.^9–11^ The latter are typically synthesized by high pressure synthesis or reductive topotactic reaction which often leads to materials with disordered anions such as AECrO_2_H (AE = Sr, Ba)^10,11^ or BaTiO_3−*x*_H_*x*_ ^12,13^ as archetypical examples. Undoubtedly, transition metal-based oxy hydrides show fascinating characteristics on their own such as magnetic ordering at elevated temperatures^10,11,14^ diffusional dynamics,^15^ good electronic^13,16^ or ionic^17^ conductivities. However, yet no single hydridic compound containing complex transition orthooxometalate anions such as tetrahedral MoO_4_^2−^ or WO_4_^2−^ anions has been reported. The reductive nature of hydrogenation reactions often requires carefully designed synthetic routes to keep the complex anions intact. In turn, only a handful mixed anionic hydrides with complex (oxo-)anions, such as aluminate hydrides^18,19^ or borate hydrides^7^ are reported to date. The combination of complex oxoanions of transition metals with hydrides has not yet been realized to date. Expanding the field of mixed anionic hydrides to complex transition metalate anions, by binding the oxygen covalently to the metal center, is expected to uncover different and potentially unforeseen and desirable material properties.

Here we report the direct synthesis, structure and electronic properties of the compounds A_3_MO_4_H (A = Rb, Cs; M = Mo, W) which are the first four representatives of the transition oxometalate hydrides. These are also the first oxide-based hydrides containing molybdenum and tungsten as transition metal. Reduction of the transition metal is avoided by an exploratory optimized synthesis route which allows to keep the transition metal with high oxidation number and the complex metalate ions intact. Moreover, covalent or coordinative interactions between the hydride and the transition metal center can be excluded in the presented compounds.

## Results and discussion

**Fig. 1:**
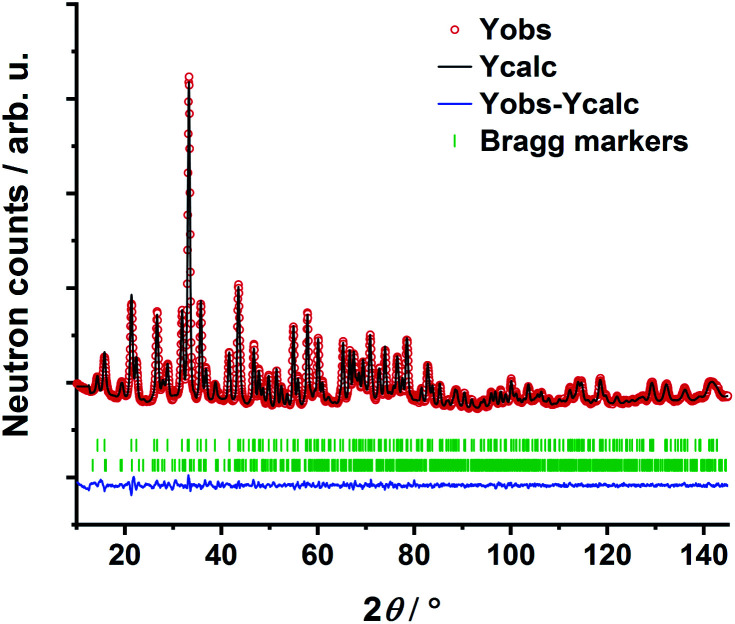
Rietveld refinement of Cs_3_MoO_4_D based on powder neutron diffraction. Bragg markers: Cs_3_MoO_4_D (top) (90.3(10) wt%); CsD (bottom) (9.7(1) wt%). *R*_p_ = 1.18%, *R*_wp_ = 1.57%, *R*_e*x*p_ = 0.83%, *R*_Bragg_ = 1.60%, *χ*^2^ = 3.59.

The transition oxometalate hydrides are synthetically accessible by a solid-state reaction under hydrogen pressure with controlled conditions. As inspired by a recent study about a novel sulfate hydride,^20^ a thermal synthesis route is applied to synthesize the herein presented compounds. In a typical synthesis, the alkaline metal A (A = Rb, Cs) is reacted with the quasi-binary oxometalate salts (A_2_MoO_4_, A_2_WO_4_) at 528 K for the molybdate hydrides and 600 K for the tungstate hydrides under an applied hydrogen pressure of 10 bar. The hydrogen pressure is required to hydrogenate the alkaline metal to form the alkaline hydride which readily reacts with the quasi-binary molybdate or tungstate salts to form the respective transition oxometalate hydrides. Mild conditions are required to avoid reduction of the transition metalate ion to the elemental transition metal or different bronzes thereof; however, too mild conditions drastically prolong the reaction time and impede phase pure synthesis or prevent the reaction as a whole. The molybdate hydrides are only accessible in a temperature window of approximately 15 K as the reduction of the molybdate ions is beginning above 535 K. The formation of the tungstate analogues occurs over a range of up to 60 K. A mechanochemical activation route as demonstrated in previous studies about new mixed anionic hydrides^7,21^ resulted in the reduction of the oxometalate ions into several different valent transition metal oxides. Short scan powder X-ray diffraction measurements of the obtained polycrystalline powders revealed diffraction patterns of new, unknown phases. Indexing of reflections from the X-ray diffraction patterns return tetragonal structures for Rb_3_MoO_4_H, Cs_3_MoO_4_H and Cs_3_WO_4_H and an orthorhombic structure for Rb_3_WO_4_H. Initial structural models were obtained by using superflip^22^ as implemented in Jana2006.^23^ Due to the very weak X-ray scattering power of hydrogen and the abundance of heavy metal atoms, we applied powder neutron diffraction of the deuterated analogues to obtain complete structural models of the newly formed phases. Due to the large bound coherent scattering length (6.671 barn) of deuterium (^2^H),^24^ the deuteride and the corresponding equal hydride positions were determined reliably, completing and enhancing the initial structural models obtained by X-ray diffraction. Subsequently, structure solution has been carried out by Rietveld-refinement of neutron diffraction data at room temperature with Fullprof.^25^ An exemplary neutron refinement plot of Cs_3_MoO_4_D is shown in Fig. 1. Structural data and all further Rietveld refinement plots obtained from X-ray and neutron diffraction data can be found in the ESI.†

The compounds Rb_3_MoO_4_D, Cs_3_MoO_4_D and Cs_3_WO_4_D crystallize isostructural in the tetragonal K_3_SO_4_F-structure type with the space group *I*4/*mcm* (140),^26^ while Rb_3_WO_4_D presumably adopts a different structure-type. The corresponding cell parameters are listed in Table 1. The building principle of all compounds is related to an A_3_BX antiperovskite-like structure. The hydride (X) occupies the octahedral site and is octahedrally coordinated by the alkaline metal (A). The larger complex anions (B) occupy the cuboctahedral voids within the [A_3_B]^+^ ReO_3_-type network. The tetragonal phases belong to the K_3_SO_4_F-structure type and show activated octahedral tilts along the *c*-direction when compared to the ideal cubic perovskite structure in *Pm*3̄*m*. The assigned glazer tilt notation is a^0^a^0^c^−^.^27^ In addition to the prototype K_3_SO_4_F,^26^ several compounds with tetrahedral complex anions are known to crystallize in this structure-type such as the selenate fluoride K_3_SeO_4_F,^28^ the oxonitrodosilicates Ln_3_[SiN_3_O]O (Ln = La, Ce, Pr),^29^ or the aluminate hydride Sr_3_AlO_4_H.^19^ A schematic of the crystal structure of the tetragonal phases can be found in the ESI in Fig. S9.†

**Table 1:** Crystallographic data of the four new compounds A_3_MO_4_D(H) (A = Rb, Cs; M = Mo, W)

	Rb_3_MoO_4_D	Cs_3_MoO_4_D	Cs_3_WO_4_D	Rb_3_WO_4_D
Space group	*I*4/*mcm* (140)	*I*4/*mcm* (140)	*I*4/*mcm* (140)	*Pbca* (61)
Phase prototype	K_3_SO_4_F	K_3_SO_4_F	K_3_SO_4_F	Own structure type
Lattice parameter (Å)	*a* = 7.8620(3)	*a* = 8.2113(2)	*a* = 8.2331(2)	*a* = 11.9262(3)
	*c* = 12.2998(5)	*c* = 12.7893(4)	*c* = 12.8289(3)	*b* = 11.3972(5)
				*c* = 11.4492(5)
Formular units (*Z*)	4	4	4	8
M–O dist. (Å)	1.766(1)	1.767(1)	1.775(1)	1.735(10)–1.784(7)
∠ (*Ø*): O–M–O, (M = Mo, W)	109.32°	109.17°	109.17°	109.42°
Glazer tilt notation	a^0^a^0^c^−^	a^0^a^0^c^−^	a^0^a^0^c^−^	a

a Due to distortions of the octahedra, the application of the Glazer-notation is not straightforwardly applicable; however, when neglecting these distortions, the same tilt-system as for the other compounds is obtained.

The Mo–O bond lengths are found to be in average 1.766 Å, while the W–O bond lengths are found to be 1.775 Å. Both agree with typical Mo–O bond lengths within the orthomolybdate ion (1.70 Å)^30^ and W–O bond lengths (1.79 Å) of orthotungstate ions.^31^ The tetrahedron angles within the complex orthometalate ions are found to have mean values in the range of 109.17–109.32° which fit closely to the ideal tetrahedron angle of 109.47°.

**Fig. 2:**
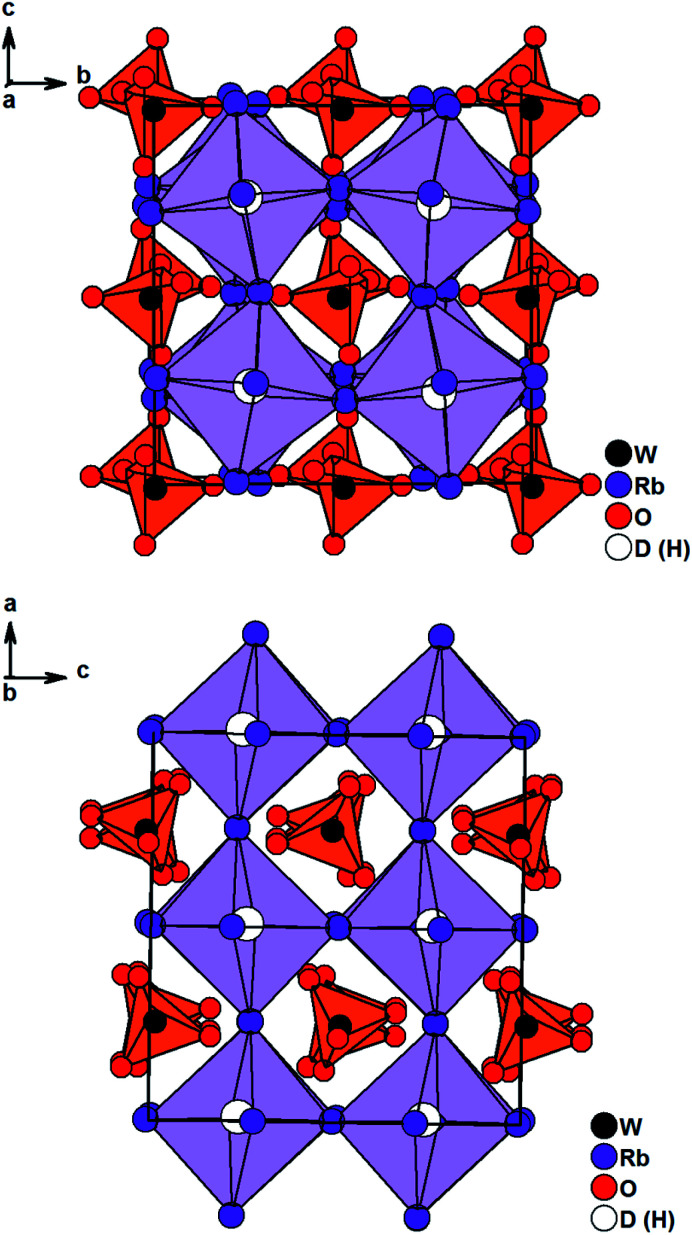
Crystal structure of Rb_3_WO_4_D along the *c*-axis (top) and the *a*-axis (bottom). Tungstate anions are depicted as orange tetrahedrons, Rb_6_D octahedrons are lilac.

Solely Rb_3_WO_4_H could not be solved in *I*4/*mcm*. Careful structural analysis based on neutron and X-ray diffraction data, delivers a new orthorhombic structure type with the space group *Pbca* (61). In this presented structure model, the Rb-built octahedrons surrounding the hydrides are distorted and tilted towards each other, most notably in the *c*-direction (Fig. 2). Also, the tungstate ions located in the cuboctahedral voids between the corner-sharing Rb_6_D octahedrons are tilted slightly towards each other in all three crystallographic directions. Overall, these slight distortions result in an antiperovskite-like structure with a pseudo tetragonal setup (*a*/*b* = 1.0464, *b*/*c* = 0.9955, *c*/*a* = 0.9600). Notably, such a distorted (anti)perovskite-like variant has not been observed this far and differs from other orthorhombic perovskite variants in the GdFeO_3_-structure type and derivatives thereof. As the Rb_6_D octahedra are unusual with Rb-positions close to special positions, several different structure solutions with varying space-groups were tested; however, no other obtained solution sufficiently converged or enhanced the herein presented model. We thus conclude the reported structural model to be the most fitting hitherto. In average the W–O bond lengths are found to be 1.77 Å, again fitting the typical W–O bond length of orthooxotungstate ions of 1.79 Å.^31^ The tetrahedron angles are found to be in average 109.42° which fits very closely to the ideal tetrahedron angle of 109.47° The Rb–D distances are found to be between 2.8529 Å and 3.0040 Å corresponding to the typical bond lengths found in ionic metal hydrides.^7,20,21,32^ Further details on the crystal structure investigations are given in the ESI,† on quoting the depository numbers CSD 2127403 (Rb_3_MoO_4_D), CSD 2127400 (Cs_3_MoO_4_D), CSD 2127401 (Cs_3_WO_4_D), CSD 2127405 (Rb_3_WO_4_D). As already stated, the cesium compounds Cs_3_MoO_4_H and Cs_3_WO_4_H are isostructural and rather unexpectedly, the structures of the rubidium based phases Rb_3_MoO_4_H and Rb_3_WO_4_H differ from one another. Due to the lanthanide contraction, molybdenum and tungsten have equal ionic radii, therefore it is expected for both compounds to be isostructural; however, when considering M–O bond lengths, the Mo–O bond length is in average about 0.01 Å shorter than the W–O bond length and thus, the molybdate ions overall have a marginal smaller total ionic radius compared to the tungstate ions. This results in slightly different packing factors which might cause the formation of different structural distortions. Interestingly, Schmitz-Dumont and Weeg observed an identical trend of the corresponding fluoride molybdates and fluoride tungstates. Even though they did not report any structural data, laboratory powder diffraction data revealed two different crystallographic set-ups for Rb_3_MoO_4_F and Rb_3_WO_4_F.^33^

To further understand the structural modifications of the antiperovskite-like structures, we calculated the Goldschmidt-tolerance factor of all four compounds. According to Goldschmidt, a compound with the general formula ABX_3_ forms the ideal cubic (anti)perovskite structure when the ionic radii have a certain ratio or simply when *t* ≈ 1.^34^ Such compounds usually adopt distorted variants if *t* differs too far from the ideal value of 1, often if *t* < 0.9 or *t* > 1.1.^34–36^ While many deviations from this trend are known, the tolerance factor is a powerful approach for rationalizing the crystal chemistry especially when applied to material series. For the here investigated systems, the tolerance factors can be calculated by considering the molybdates and tungstates as complex ions, applying the formula below:^35–37^
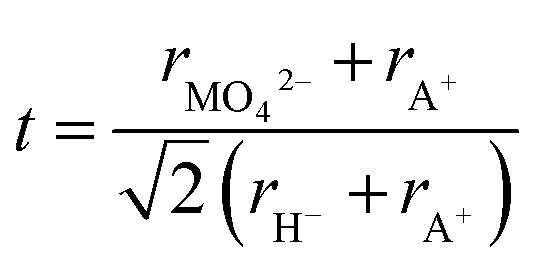


For details on the determination of ionic radii of MO_4_^2−^ and H^−^ see ESI.†

As seen in Table 2, the determined tolerance factors all deviate from the ideal value of *t* ≈ 1; however, they fit closely to the determined value of the phase prototype K_3_SO_4_F, with Cs_3_MoO_4_H having the best fitting value of 1.11. By a further look at the tolerance factors, it is recognizable that Rb_3_WO_4_H deviates the most from the phase prototype and the related tetragonal phases, with a calculated tolerance factor of 1.14. As suggested by the Goldschmidt-factor, the Rb^+^ ion in this structure might be just too small in relation to the large complex WO_4_^2−^ anion to stabilize Rb_3_WO_4_H in a less distorted structure when compared to the other compounds reported in this work. The tolerance factor deviates even more from 1 in Na_3_SO_4_H which represents a further antiperovskite-like hydride (*P*4/*nmm*, Ag_3_CrO_4_Cl-type).^20^ Compared to the structure types presented in this work, the assembly is different in Na_3_SO_4_H as the alkaline (Na^+^) ions are now considerably smaller than the hydride ions. In turn, the sulfate anions demand less space within the cuboctrahedral voids in relation to the larger oxometalate anions. This overall results in another tetragonal structure with only distorted but not tilted (Na_6_D) octahedra.

**Table 2:** Determined Goldschmidt-tolerance factors

Compound	Tolerance factor *t*
Rb_3_MoO_4_H	1.12
Cs_3_MoO_4_H	1.11
Rb_3_WO_4_H	1.14
Cs_3_WO_4_H	1.12
K_3_SO_4_F	1.09
Na_3_SO_4_H	1.15

### MAS NMR spectroscopy

Structure analysis based on X-ray and neutron diffraction is complemented by magic angle spinning nuclear magnetic resonance (MAS NMR) to obtain information on the atomic level. Especially ^1^H and ^2^H MAS NMR have proven to be a powerful tool to confirm the presence of hydride ions.^7,8,20,21,38^

^1^H is the most receptive nuclear spin, however, the ^2^H spin is superior as the spectra are not affected by any other present hydrogen containing material like impurities from the probe background or from synthesis.

**Fig. 3:**
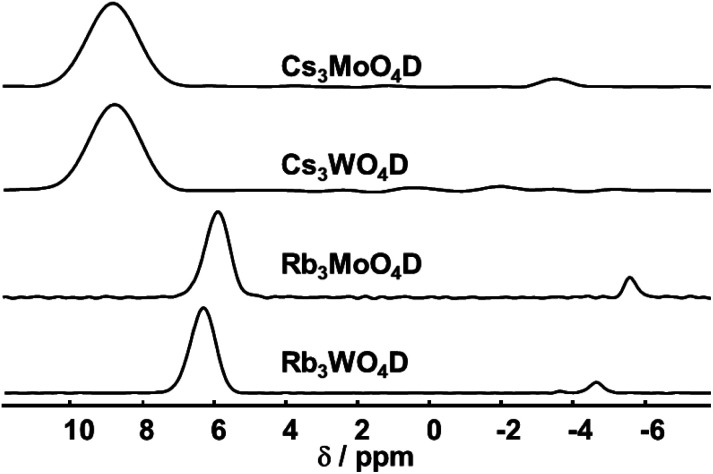
^2^H MAS NMR spectra of the four compounds (isotropic region only). The spectra were acquired at room temperature with a spinning frequency of 5 kHz and a magnetic field *B*_0_ = 17.6 T.

The ^2^H MAS spectra of the four samples are summarized in Fig. 3. Corresponding ^1^H spectra show the same signals with quasi identical shifts, yet contain additionally other signals originating from the rotor cap or other external impurities. All obtained ^2^H MAS NMR spectra contain one dominant signal that is assigned to the parent material. Additionally, in all samples a minor signal with a small linewidth at negative chemical shift is present. This signal originates from hydrides covalently bound to transition elements, typically showing negative shifts.^39^ In the case of Rb_3_WO_4_D and Cs_3_MoO_4_D a quadrupolar pattern indicative of a covalent bond is seen. Since these signals contribute only to a minor amount besides the main signal, a more detailed analysis was not done.

Both rubidium and both cesium containing samples show each very similar shifts, about 6.0–6.4 ppm for rubidium and 9.8 ppm for cesium. The higher shift for cesium is expected following the trend of the size of alkali metal hydrides and corresponding shifts in the simple hydrides (LiH: 2.9 ppm, NaH: 3.6 ppm, KH: 4.7 ppm).^40^ DFT-PBE calculations of the chemical shift of the ^1^H nucleus support the experimental findings. The shifts were calculated to be 6.4 ppm for Cs_3_MoO_4_H, 6.2 ppm for Cs_3_WO_4_H and 5.5 ppm for both rubidium compounds in reference to SiMe_4_. While the calculated shifts of the cesium compounds differ compared to the experimental findings, the trend of the higher homologues to be downfield shifted is reproduced. In the case of cesium, the mismatching downfield shift might be due to the spin–orbit heavy-atom effect on the light-atom, where the heavy cesium atom has a deshielding effect of on the neighbouring H atom.^41^ Spin–orbit coupling effects have not been taken into account in the present calculations.

Overall, the chemical shifts were found to be in the region typical for inorganic salt-like hydrides.^7,8,20,21,38,40^ In combination with DFT calculations, ^2^H MAS NMR proves the abundance of hydrides within the crystal lattice.

Further evidence of the hydride abundance is provided by simple elemental analysis. Here, the experimental determined weight percentage of hydrogen is determined to be close to the theoretical values in all four compounds. The simultaneous abundance of either tungsten or molybdenum is additionally determined and underlines the abundance of both hydride anions next to tungstate and molybdate ions. The elemental analysis reports can be seen in the ESI.†

**Fig. 4:**
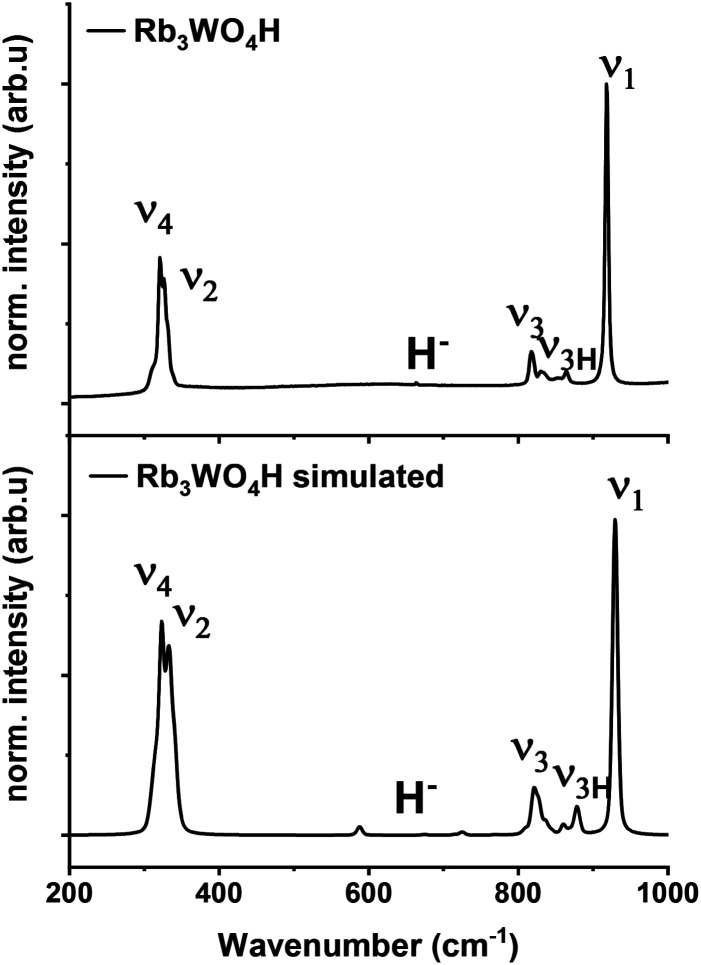
Experimental Raman spectrum of Rb_3_WO_4_H (top) and simulated Raman spectrum of Rb_3_WO_4_H (bottom, DFT-PBE0 method).

Raman spectroscopy is used to verify the abundance of complex tetrahedral (*ortho*)anions through the presence of their typical stretching and bending modes. The experimental spectra were additionally compared to simulated spectra obtained by density functional theory (DFT-PBE0) calculations of the hydridic species (see ESI† for the computational details). As can be seen in Fig. 4 and S25–S27† the obtained Raman spectra are in good agreement with the simulated spectra. All Raman-active vibrational modes, *ν*_1_ to *ν*_4_, are observed in the expected wavenumber regions with the predicted intensity, confirming the abundance of the complex orthometalate anions and supporting in overall the structural models. The Raman spectra also differ from the corresponding Raman spectra of the binary oxometalate salts. The respective spectra, due to the lower orthorhombic symmetry of the starting materials, show a splitting of the *ν*_3_ mode and overlapping *ν*_2_ and *ν*_4_ modes. This deviates from the spectra of the newly formed phases where the *ν*_2_ and *ν*_4_ modes appear noticeable distant to each other and the *ν*_3_ mode does not show splitting.^42^ As the structure of Rb_3_WO_4_H differs from the structure of the tetragonal phases, its Raman spectrum shows a slightly different Raman spectrum (Fig. 4). In addition to the vibrational modes of the tungstate anions, vibrational modes of the tungstate anions coupled to hydride modes (*ν*_3H_) are seen at about 850–900 cm^−1^ as predicted in the simulated spectrum. This again confirms the abundance of hydride ions and supports the structural model obtained by neutron diffraction. By comparison with the Raman spectrum of Rb_2_WO_4_, it is apparent that in this case the *ν*_3_ modes are more distinctly split and the *ν*_3H_ modes are missing. Similarly for the *ν*_2_ and *ν*_4_ modes that show more recognizable and pronounced bending modes, not seen in the Raman spectrum of Rb_3_WO_4_H.^43^ This overall affirms the successful formation of a new phase.

Fig. S19, S21 and S23† show the calculated electronic band structures and density of states of the tetragonal phases crystallizing in the K_3_SO_4_F-structure. All three compounds can be classified as wide band gap semiconductors with direct band gaps of approximately 3.2 eV (Cs_3_MoO_4_H), 3.4 eV (Rb_3_MoO_4_H) and 3.8 eV (Cs_3_WO_4_H). All three calculated band structures show similar features where hydride is dominating the topmost valence band with only minor contributions from rubidium or cesium. Due to the polarizability of hydride and the strong covalent character of the hydride ion, the topology of the band structure is directly influenced by the hydride ion and is directly responsible for the direct band gap and thus the semiconducting character of the tetragonal compounds. These findings reflect and are in line with previous studies of inorganic salt like hydrides where hydride is always predicted to dominate the topmost valence band.^6–8,20,44^ UV/Vis absorption spectroscopy and the resulting Tauc-plots confirm the direct band gaps and are close to the estimated band gap value which underlines the direct influence of the hydride ion regarding the direct band gap. As the compounds are isostructural, a band gap tuning might be possible by the synthesis of mixed cationic or mixed tungstate/molybdate solid-solutions.

**Fig. 5:**
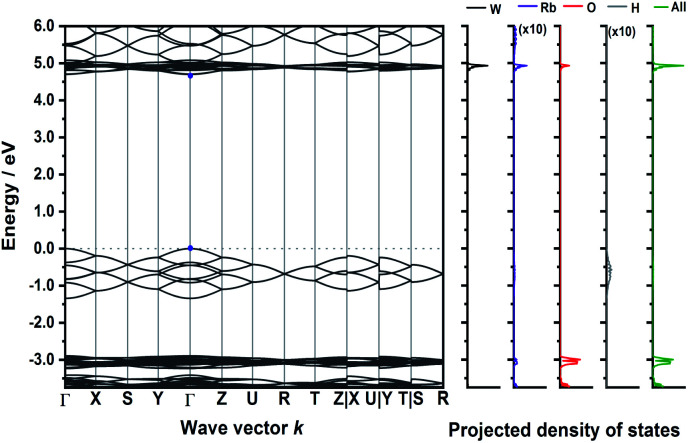
Electronic band structure of Rb_3_WO_4_H and projected density of states (DFT-PBE0). The band paths in the reciprocal space have been determined by the Seek-path webservice.^45^ The DOS of Rb and H are enhanced by a factor of ten for better visibility.

The calculated band structure of Rb_3_WO_4_H (Fig. 5) is very peculiar and the valence bands are dominated by the hydride states. At the *Γ*-point, all eight hydride bands are non-degenerate, while at the *R*-point all states are degenerate. In this crystal structure, the hydrides form a quasi-cubic arrangement, resulting in slightly unequal paths within the reciprocal space. Even though a relatively large band gap of approx. 4.6 eV is estimated, again a direct transition is predicted.

Interestingly, in all four calculated band structures the states arising from the hydride ions are located between states arising from the complex transition metalate ion. An initial approach for the design of direct semiconductors might target a modification of this band structure.

## Conclusions

In summary, we hereby provide the groundwork for a potentially broad class of materials: the transition oxometalate hydrides. The four compounds A_3_MO_4_H (A = Rb, Cs; M = Mo, W) are accessible *via* a sophisticated thermal synthesis route and are introduced as the first representatives of this new class. To the best of our knowledge, such a combination of transition metal anions with hydride ions has not been realized this far. The unprecedented abundance of hydrides next to complex oxoanions within single structures is proven by several analytical methods including neutron diffraction, Raman spectroscopy, MAS NMR spectroscopy, elemental analysis and beyond that supported by quantum chemical calculations. The four compounds show interesting electronic and structural features. While the compounds of the tetrahedral class are direct and wide band gap semiconductors, Rb_3_WO_4_H shows a very dispersive, peculiar valence band structure dominated by hydride states arising from the pseudo cubic arrangement of the hydride ions within the orthorhombic crystal structure. Overall, these findings demonstrate a pathway to hitherto unexplored anion combinations and open the door for further anion combinations containing other complex transition metalate ions like *e.g.* (di)chromates or orthovanadates. Likewise, these compounds may act as chemical templates for new, more complex structures such as combinations of hydrides with polymetalate ions.

## Data availability

Full experimental details and further data supporting the research are provided in the ESI.†

## Author contributions

A. M., G. K. and N. K. coordinated the research and wrote the main parts of the manuscript, A. M. and A. S. performed the syntheses, M. B. performed the NMR experiments, A. M. and A. J. K. performed DFT calculations, C. R. collected the neutron diffraction data, N. K. acquired funding and administrated the project. All authors commented on the paper.

## Conflicts of interest

The authors declare no conflict of interest.

## Supplementary Material

SC-013-D2SC01861F-s001

SC-013-D2SC01861F-s002
