# Supplementary material for: Expanding the hydride chemistry: antiperovskites A3MO4H (A = Rb, Cs; M = Mo, W) introducing the transition oxometalate hydrides
Source: Chem Sci. 2022 May 25;13(26):7773–9. doi: 10.1039/d2sc01861f (PMC9258318; doi:10.1039/d2sc01861f)
Supplement: SC-013-D2SC01861F-s001 [file SC-013-D2SC01861F-s001.pdf]

Electronic Supplementary Material (ESI) for Chemical Science.
This journal is © The Royal Society of Chemistry 2022

Expanding the hydride chemistry: Antiperovskites A_3MO_4H (A = Rb, Cs; M = Mo, W) introducing the transition oxometalate hydrides

Alexander Mutschke, Annika Schulz, Dr. Marko Bertmer, Dr. Clemens Ritter, Prof. Dr. Antti J. Karttunen, Dr. Gregor Kieslich, Dr. Nathalie Kunkel

Table of Content

1 Experimental section 3
1.1 Synthesis 3
1.2 Powder X-ray diffraction 3
1.3 Neutron diffraction 3
1.4 Rietveld Refinement 3
1.5 Raman spectroscopy 3
1.6 UV/VIS absorption spectroscopy 3
1.7 Elemental analysis 4
1.8 Solid-state NMR 4
1.9 Quantum chemical calculations 4
2 Results and discission 5
2.1 Structural analysis 5
2.1.1 Neutron diffraction 5
2.1.2 Supplementary X-ray diffraction data (Hydridic samples) 13
2.2 Ionic radii for determination of the Goldschmidt tolerance factors 17
2.3 Crystal structure of the K_3SO_4F-structure type 18
2.4 Difference Fourier maps 19
2.5 ^{2}H MAS NMR Spectroscopy 23
2.6 Calculated electronic band structures and UV/Vis Tauc plots 25
2.7 Vibrational Spectroscopy (experimental and DFT-PBE0) 28
2.8 Elemental Analysis 32
2.9 Optimized crystal structures as CIF (DFT-PBE0) 33
3 Literature 36

1 Experimental section

1.1 Synthesis

Due to the air and moisture sensitivity of the reactants and the resulting products, all preparative and analytical operations were carried out in argon-filled gloveboxes. The O_2 and H_2O concentrations were kept below 0.5 ppm respectively.

The transition oxometalate hydrides A_3MO_4H (*A* = Rb, Cs; *M* = Mo, W) can be synthesized by the solid-state reaction of in-situ formed alkaline hydride with the quasi-binary oxometalate salts A_2MO_4. (*A* = Rb, Cs; *M* = Mo, W)

The binary molybdate salts were synthesized by a solid-state reaction of MoO_3 (*Alfa Aesar*, 99.5%) with A_2CO_3. (Rb_2CO_3: *Chempur*, 99.9%; Cs_2CO_3: *Alfa Aesar*, ≥ 99%) in stoichiometric ratios. Thereto, the two powders were grinded thoroughly in an agate mortar, transferred in a corundum crucible and heated at 620 °C for 15h. If residual reactants were observed in short scan X-ray diffraction patterns, the product was reground and annealed again with the same temperature program.

The tungstate salts Rb_2WO_4 (*Chempur*, 99.9%), Cs_2WO_4, (*Alfa Aesar*, 99%), were purchased commercially. Before use, the binary oxometalate salts were dried under dynamic vacuum at 200 °C for 24 h, twice, to remove any traces of moisture.

To synthesize the transition oxometalate hydrides, the alkaline metal (Rb: *Alfa Aesar*, 99.75%, Cs: *Alfa Aesar,* 99.8%) is mixed thoroughly in an agate mortar with the dry binary transition metalate salt for several minutes. The reactive mixture is then heated in a self-made autoclave made of a hydrogen-resistant alloy (*Inconel Böhler 718*) at 255 °C (if *M* = Mo) or at 330 °C (if *M* = W) and applied hydrogen (*Westfalen AG*, 99.9%)/deuterium (*AirLiquide*, 99.9%) pressure of 10 bars. During the reaction, the alkaline metal is hydrogenated/deuterated to form the alkaline hydride/deuteride (RbH/D or CsH/D) which readily reacts with the binary transition metalate salt to form the products A_3MO_4H/D.

1.2 Powder X-ray diffraction

Powder X-ray diffraction data were recorded on a Stoe STADI-P in transmission geometry with either Cu-$K_{\alpha 1}$ (λ = 1.54059 Å) or with Mo-$K_{\alpha 1}$ (λ = 0.70930 Å) radiation. Both setups are equipped with a curved Ge-monochromator (111) and a Dectris Mythen DCS 1K solid-state detector. To avoid decomposition of the samples during measurements, the powders were placed in sealed in glass capillaries (Ø 0.3mm, 0.01mm wall thickness). Due to severe X-ray absorption at Cu-$K_{\alpha 1}$ radiation and fluorescence at Mo-$K_{\alpha 1}$ radiation, Rb_3WO_4H/D was sealed within Ø 0.1 mm glass capillaries to minimize these effects.

1.3 Neutron diffraction

Neutron powder diffraction data of all four deuterium analogue compounds were recorded at room temperature at the two-axis high-resolution powder neutron diffractometer D2B at the Institut Laue-Langevin (ILL), Grenoble using a wavelength of 1.594 Å. Each powder diffraction pattern was recorded over the course of 5h. For the measurement ~4 g of the sample were enclosed in 9 mm vanadium cylinders and sealed airtight with indium wires

1.4 Rietveld Refinement

Crystal structure refinement of X-ray diffraction and neutron diffraction data was done using the program package FullProf[1] with the Rietveld method and the fundamental parameter approach. Profiles were fitted using pseudo-Voigt functions. The zero shift, cell parameters, three form factors (Caglioti parameters U, V, W), either two (neutron diffraction data) or four (X-ray diffraction data) asymmetry parameters, atomic positions, isotropic and if applicable, anisotropic thermal displacement parameters were refined. The background correction was done with linear interpolations between refinable background points.

1.5 Raman spectroscopy

Raman spectra were recorded on the polycrystalline samples sealed in glass capillaries (Ø 0.3mm, 0.1mm wall thickness) on a Renishaw inVia Reflex Raman System equipped with a CCD detector. The wavelength of the laser was λ = 532 nm and the resulting spectra were recorded in the range of 100-1200 cm^{-1}.

1.6 UV/VIS absorption spectroscopy

UV-Vis absorption spectra were recorded on a *Shimadzu* UV-3600 Plus UV-Vis-NIR spectrophotometer. Thin films of the polycrystalline samples were placed within two quartz glass slides under Argon atmosphere. The glass slides were sealed airtight with vacuum grease for measurement. Direct and possible indirect bandgaps were determined from the materials absorption *α* obtained by the acquired solid-state UV-Vis absorption spectra.

Applied formula:

$$E\,(\text{eV}) = \frac{1240}{\lambda\,(\text{nm})}\,(\alpha \text{h}\upsilon)^{\frac{1}{r}}$$

r is set to ½ for an allowed, direct transition (direct bandgap).[2]

1.7 Elemental analysis

Elemental analysis to determine and prove the hydrogen abundance of the hydridic samples has been conducted by CHNS analysis on a Vario El microanalyzer. Thereto, roughly 3 mg of the analyzed substance was packed within tin crucibles and folded within further tin crucibles to keep the samples air-tight until measurement.
Elemental analysis to determine the tungsten and molybdenum content has been conducted via alkaline pulping of the samples and consecutive photometric analysis. Thereto, 5 mg (molybdenum determination) or 12 mg (tungsten determination) of the respective samples were packed within aluminium boats beforehand and sealed airtight until measurement. The tungsten determination has slight deviations (~ 1%) due to strong hygroscopic properties of the tungstate salts.

1.8 Solid-state NMR

Measurements were taken at a magnetic field strength of 17.6 T at frequencies of 748.43 MHz and 114.88 MHz for ^1H and ^2H, respectively. Magic angle spinning (MAS) was done at rotation frequencies of 12 and 5 kHz. Single pulse excitation with recycle delays of 60 s and 2000 s for ^1H and ^2H were used, respectively. Recycle delays were chosen to be long enough for full relaxation; exact spin-lattice relaxation measurements (T_1) were not done. In case of ^2H full relaxation might not have been reached for the parent components. The transition metal hydrides with negative shifts relax much faster. Spectra are referenced indirectly to TMS or d-TMS via the ^1H shift of a sample of PDMS (0.07 ppm to TMS).

1.9 Quantum chemical calculations

The geometries, electronic properties, and vibrational properties of the four compounds A_3MO_4H (A = Rb, Cs; M = Mo, W) were studied with the CRYSTAL17 program package[3]. PBE0 hybrid density functional method and Gaussian-type basis sets were used.[4,5] The basis sets for Rb, Cs, Mo, W, O, and H have been previously derived from the molecular Karlsruhe def2 basis sets.[6]. Polarized triple-zeta-valence (TZVP) basis sets were used for Mo, W, O, and H polarized split-valence basis set for Rb and Cs (SVP)[7]. The reciprocal space was sampled using a 4×4×4 Monkhorst-Pack-type k-mesh for the tetragonal structures and 3×3×3 Monkhorst-Pack-type k-mesh for the orthorhombic structure of Rb_3WO_4H[8]. Tightened tolerance factors (TOLINTEG) of 8, 8, 8, 8 and 16 were used for the evaluation of the Coulomb and exchange integrals. Both the atomic positions and lattice constants were fully optimized within the constraints imposed by the space group symmetry. The optimized lattice parameters a and c for the tetragonal phases differed from the experimental parameters by -0.1% and +1.2% for Rb_3MoO_4H, +0.1% and +1.1% for Cs_3MoO_4H, +0.1% and +0.1% for Cs_3WO_4H respectively. The optimized and experimental lattice parameters *a*, *b* and *c* for the orthorhombic phase Rb_3WO_4H differed +0.4%, +0.7%, and +0.5%, respectively. Raman intensities and corresponding spectra were obtained using the computational schemes implemented in CRYSTAL.[9] The optimized structures of the tetragonal structures were confirmed to be true local minima with no imaginary frequencies. The optimized structure of Rb_3WO_4H showed a very small imaginary frequency of –16.4 cm^{-1}, which did not disappear when the structure was distorted along the mode (tungstate deformation vibration, B_{1u}). We thus expect this mode to arise for example from small numerical inaccuracies in the numerical integration of the exchange-correlation functional. The Raman intensities have been calculated for a polycrystalline powder sample (total isotropic intensity in arbitrary units). For the simulation of the Raman spectra the temperature and laser wavelength were set to values corresponding to the experimental setup (T = 298.15 K, λ = 532 nm). The peak profile of the final spectra were simulated a using pseudo-Voigt peak profile (50:50, Lorentzian: Gaussian) and FWHM of 8 $cm^{-1.}$

The solid-state NMR shielding tensors were calculated with the DFT-PBE method[4], using the CASTEP program package and the GIPAW formalism as implemented in CASTEP-NMR[10]. Ultrasoft pseudopotentials generated with the on-the-fly scheme and a plane-wave basis were applied[11]. The kinetic energy cutoff was set to 630 eV for A_3MO_4H (A = Rb, Cs; M = Mo, W), while a cutoff of 450 eV was used for RbH and CsH. The reciprocal space was sampled using the following Monkhorst-Pack-type *k*-meshes: 4×4×4 for tetragonal A_3MO_4H structures, 2×2×2 for Rb_3WO_4H, and 8×8×8 for RbH, CsH. The NMR shielding tensors of A_3MO_4H were calculated at both the experimental geometry and DFT-PBE optimized geometry, but the resulting ^1H NMR shifts are practically similar at both geometries. In the geometry optimization, both the lattice parameters and atomic positions were fully optimized with a total energy convergence criterion of 0.5 x 10^{-6} eV/atom. Molecular $SiMe_4$ was used as a reference system for calculating the ^1H NMR shifts. The calculations on $SiMe_4$ were carried out in a primitive cubic cell (a = 15 Å) using a plane-wave basis set cut-off of 700 eV and Γ-point for reciprocal space sampling. The structure of the $SiMe_4$ molecule was relaxed within the T_d point group. The isotropic ^1H shielding of $SiMe_4$ is 31.01 ppm.

2 Results and discussion

2.1 Structural analysis

2.1.1 Neutron diffraction

Rb_3MoO_4D (RT)

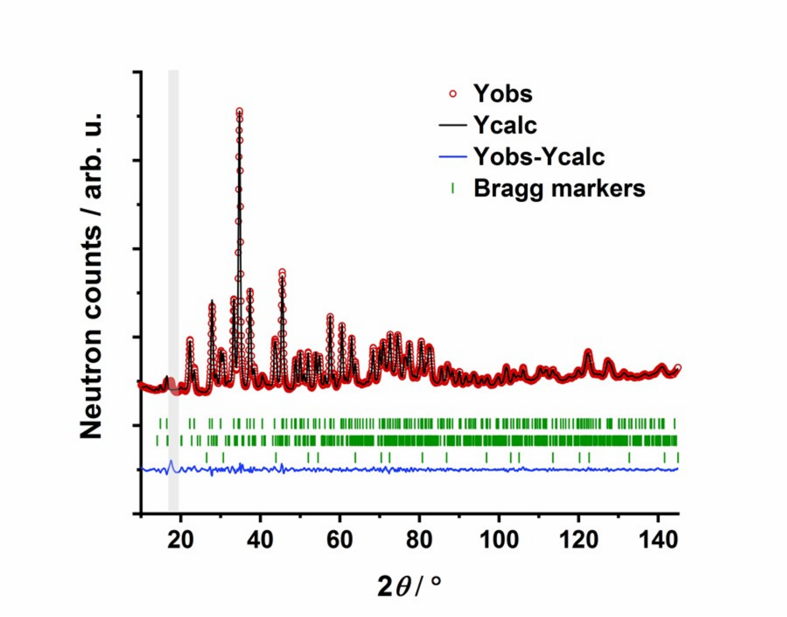

Figure S1 Rietveld refinement plot of Rb_3MoO_4D (*I4/mcm*, 140) at room temperature based on neutron diffraction data. The grey area was cut out during refinement as this reflection was not assignable. Bragg markers from top to bottom: Rb_3MoO_4D (89.3(7) wt.%), Rb_2MoO_4 (*Pnam*) (6.2(3) wt.%), RbD ($Fm\bar{3}m$) (4.6(1) wt.%)

Table S1. Rietveld refinement paramaters of Rb_3MoO_4D determined from neutron diffraction data at room temperature with the program Fullprof[1]

**Rb_3MoO_4D**	
Space group	*I4/mcm* (140)
Calculated density [g/cm^{-3}]	3.655
Step scan increment	0.05
2*θ* range (°)	10 - 158
Wavelength (Å)	1.594
Number of profile points	2960
Temperature (K)	298
Program	Fullprof
Shape parameter η	0.539 (10)
Caglioti parameters (*U, V, W*)	*U* = 0.484(17)
	V = -0.510(19)
	W = 0.395(1)
Number of reflections	230
Number of refined parameters	112
R_{Bragg}	2.84%
R_p	1.49%
R_{wp}	2.03%
R_{exp}	0.65%
Goodness of fit (X^2)	9.60

Table S2. Crystallographic data on Rb_3MoO_4D determined from neutron diffraction data at room temperature

**Cell parameters**<br>a = 7.8620(3) Å, c = 12.2998(5) Å; a/b=1.00, b/c= 0.6392<br>V = 760.26(5) Å^3					
**Atom**	**Wyckoff position**	**Site**	**x/a**	**y/b**	**z/c**
Mo	4b	-42*m*	0	½	¼
Rb1	8h	*m*2*m*	0.19049(13)	x+½	0
Rb2	4a	422	0	0	¼
O1	16l	*m*	0.12924(13)	x+½	0.66646(17)
D1	4c	4/*m*	0	0	0

Table S3. Anisotropic displacement parameters of Rb_3MoO_4D determined from neutron diffraction data

**Anisotropic displacement parameters [Å²]**						
**Atom**	**U_{11}**	**U_{22}**	**U_{33}**	**U_{12}**	**U_{13}**	**U_{23}**
Mo1	0.0227(9)	0.0227(9)	0.0095(14)	0.00000	0.00000	0.00000
Rb1	0.0298(7)	0.0298(7)	0.0267(11)	-0.0054(9)	0.00000	0.00000
Rb2	0.0357(11)	0.0357(11)	0.0399(18)	0.00000	0.00000	0.00000
O1	0.0581(10)	0.0581(10)	0.0450(11)	-0.0184(10)	0.0222(6)	0.0222(6)
D1	0.0363(10)	0.0363(10)	0.0565(20)	0.00000	0.00000	0.00000

Table S4. Selected interatomic distances and tetrahedron angles in Rb_3MoO_4D determined from neutron diffraction data

**Atom 1**	**Atom 2**	**Distance (Å)**	
Mo1	O1	1.7665(15)	
Rb1	D1	2.8573(10)	
Rb2		3.0749(12)	
Tetrahedron angle			
Atom 1	Atom 2	Atom 3	Angle
Mo1	O1	O1	108.865(49)
	O1	O1	109.775(58)
		Ø tetrahedron angle	109.32

Cs_3MoO_4D (RT)

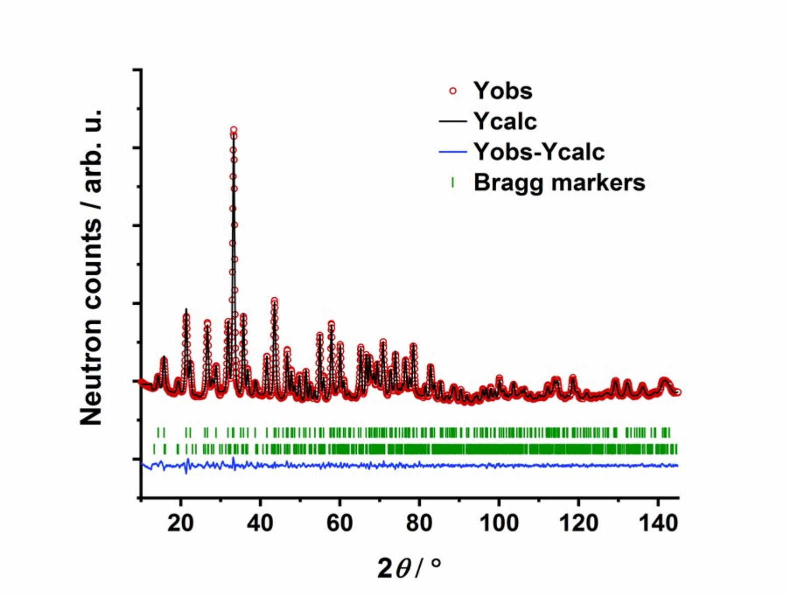

Figure S2 Rietveld refinement plot of Cs_3MoO_4D (*I4/mcm*, 140) at room temperature based on neutron diffraction data. Bragg markers from top to bottom: Cs_3MoO_4D (90.3 (10) wt.%), Cs_2MoO_4 (*Pnma*) (9.7(1) wt.%)

Table S5. Rietveld refinement parameters of Cs_3MoO_4D determined from neutron diffraction data at room temperature with the program Fullprof[1]

**Cs_3MoO_4D**	
Space group	*I4/mcm* (140)
Calculated density [g/cm^{-3}]	4.319
Step scan increment	0.05
2θ range (°)	10 - 158
Wavelength (Å)	1.594
Number of profile points	2960
Temperature (K)	298
Program	Fullprof
Shape parameter η	0.541(12)
Caglioti parameters (*U, V, W*)	*U* = 0.352(10)
	V = -0.497(13)
	W = 0.393(5)
Number of reflections	258
Number of refined parameters	98
R_{Bragg}	1.60%
R_p	1.18%
R_{wp}	1.57%
R_{exp}	0.83%
Goodness of fit (X^2)	3.59

Table S6. Crystallographic data on Cs_3MoO_4D determined from neutron diffraction data at room temperature

Cell parameters
a = 8.2113(2) Å, c = 12.7893(4) Å; a/b=1.00, b/c= 0.6420
V = 833.70(4) Å^3

Atom	Wyckoff position	Site	x/a	y/b	z/c
Mo	4b	-42*m*	0	½	¼
Cs1	8h	*m2m*	0.18705(16)	x+½	0
Cs2	4a	422	0	0	¼
O1	16l	*m*	0.12348(11)	x+½	0.66890(11)
D1	4c	4/*m*	0	0	0

Table S7. Anisotropic displacement parameters of Cs_3MoO_4D determined from neutron diffraction data

**Anisotropic displacement parameters [Å²]**						
**Atom**	$\mathbf{U_{11}}$	$\mathbf{U_{22}}$	$\mathbf{U_{33}}$	$\mathbf{U_{12}}$	$\mathbf{U_{13}}$	$\mathbf{U_{23}}$
Mo1	0.0209(8)	0.0209(8)	0.0154(12)	0.00000	0.00000	0.00000
Cs1	0.0275(7)	0.0275(7)	0.0272(13)	-0.0032(9)	0.00000	0.00000
Cs2	0.0308(10)	0.0308(10)	0.0304(18)	0.00000	0.00000	0.00000
O1	0.0421(7)	0.0421(7)	0.0376(9)	-0.0162(9)	0.0100(5)	0.0100(5)
D1	0.0487(11)	0.0487(11)	0.063(2)	0.00000	0.00000	0.00000

Table S8. Selected interatomic distances and tetrahedron angles in Cs_3MoO_4D determined from neutron diffraction data

**Atom 1**	**Atom 2**	**Distance (Å)**	
Mo1	O1	1.7697(11)	
Cs1	D1	2.9938(13)	
Cs2		3.1973(1)	
Tetrahedron angle			
Atom 1	Atom 2	Atom 3	Angle
Mo1	O1	O1	110.090(48)
	O1	O1	108.240(43)
		Ø tetrahedron angle	109.165

Cs_3WO_4D (RT)

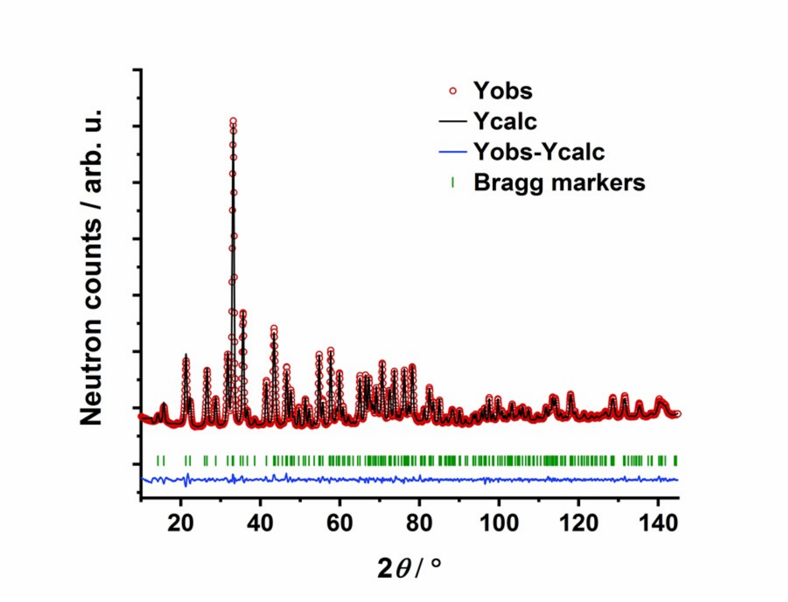

Figure S3 Rietveld refinement plot of Cs_3WO_4D (*I4/mcm*, 140) at room temperature based on neutron diffraction data. Bragg markers Cs_3WO_4D.

Table S9. Rietveld refinement parameters of Cs_3WO_4D determined from neutron diffraction data at room temperature with the program Fullprof[1]

Cs_3WO_4D	
Space group	*I4/mcm* (140)
Calculated density [g/cm^{-3}]	4.954
Step scan increment	0.05
2θ range (°)	10 - 158
Wavelength (Å)	1.594
Number of profile points	2960
Temperature (K)	298
Program	Fullprof
Shape parameter η	0.478(10)
Caglioti parameters (*U, V, W*)	U = 0.263(6)
	V = -0.474(10)
	W = 0.386(4)
Number of reflections	260
Number of refined parameters	73
R_{Bragg}	2.03%
R_p	1.53%
R_{wp}	2.04%
R_{exp}	0.87%
Goodness of fit (X^2)	5.46

Table S10. Crystallographic data on Cs_3WO_4D determined from neutron diffraction data at room temperature

Cell parameters
a = 8.2331(2) Å, c = 12.8289(3) Å; a/b=1.00, b/c= 0.6418
V = 869.58(4) Å^3

Atom	Wyckoff position	Site	x/a	y/b	z/c
W	4b	-42*m*	0	½	¼
Cs1	8h	*m*2*m*	0.18917(12)	x+½	0
Cs2	4a	422	0	0	¼
O1	16l	*m*	0.12355(9)	x+½	0.66889(9)
D1	4c	4/*m*	0	0	0

Table S11. Anisotropic displacement parameters of Cs_3WO_4D determined from neutron diffraction data

**Anisotropic displacement parameters [Å²]**						
**Atom**	**U_{11}**	**U_{22}**	**U_{33}**	**U_{12}**	**U_{13}**	**U_{23}**
W1	0.0249(8)	0.0249(8)	0.0143(12)	0.00000	0.00000	0.00000
Cs1	0.0279(6)	0.0279(6)	0.0299(10)	0.0023(7)	0.00000	0.00000
Cs2	0.0320(9)	0.0320(9)	0.0322(14)	0.00000	0.00000	0.00000
O1	0.0484(6)	0.0484(6)	0.0417(7)	-0.0184(7)	0.0132(4)	0.0132(4)
D1	0.0405(8)	0.0405(8)	0.0562(15)	0.00000	0.00000	0.00000

Table S12. Selected interatomic distances and tetrahedron angles in Cs_3WO_4D determined from neutron diffraction data

**Atom 1**	**Atom 2**	**Distance (Å)**	
W1	O1	1.7754(9)	
Cs1	D1	2.9958(10)	
Cs2		3.2072(1)	
Tetrahedron angle			
Atom 1	Atom 2	Atom 3	Angle
W1	O1	O1	110.09(4)
	O1	O1	108.240(35)
		Ø tetrahedron angle	109.17

Rb_3WO_4D (RT)

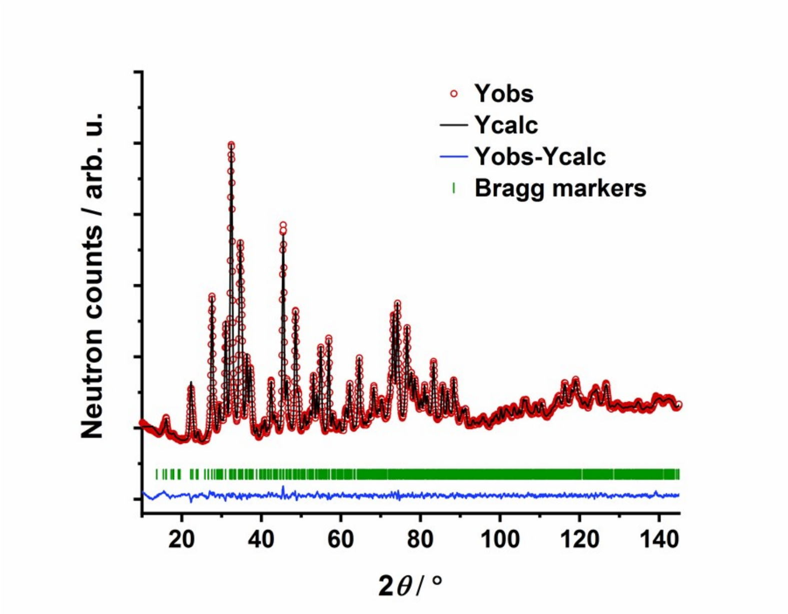

Figure S4 Rietveld refinement plot of Rb_3WO_4D (*Pbca*, 61) at room temperature based on neutron diffraction data. Bragg markers Rb_3WO_4D.

Table S13. Rietveld refinement paramaters of Rb_3WO_4D determined from neutron diffraction data at room temperature with the program Fullprof[1]

**Rb_3WO_4D**	
Space group	*Pbca* (61)
Calculated density [g/cm^{-3}]	4.322
Step scan increment	0.05
2θ range (°)	10 - 158
Wavelength (Å)	1.594
Number of profile points	2960
Temperature (K)	298 K
Program	Fullprof
Shape parameter η	0.540(10)
Caglioti parameters (*U, V, W*)	*U* = 0.348(11)<br>*V* = -0.527(14)<br>*W* = 0.405(5)
Number of reflections	1610
Number of refined parameters	140
R_{Bragg}	1.60%
R_p	1.08%
R_{wp}	1.44%
R_{exp}	0.86%
Goodness of fit (X^2)	2.77

Table S14. Crystallographic data on Rb_3WO_4D determined from neutron diffraction data at room temperature

Cell parameters
a = 11.9262(3) Å, b = 11.3972(5) Å, c = 11.4492(5) Å; a/b=1.0464 b/c=0.9955 c/a=0.9600
V= 1556.24(10)Å^3

Atom	Wyckoff position	Site	x/a	y/b	z/c
W1	8c	1	0.7464(7)	0.0054(4)	-0.0149(4)
Rb1	8c	1	0.7464(4)	0.2334(4)	0.7289(3)
Rb2	8c	1	-0.0009(4)	0.7822(4)	-0.0062(5)
Rb3	8c	1	0.0003(4)	-0.0078(4)	0.7188(3)
O1	8c	1	0.8660(5)	-0.0224(5)	0.0730(5)
O2	8c	1	0.7823(5)	-0.0226(5)	0.8365(4)
O3	8c	1	0.7173(5)	0.1588(4)	-0.0086(5)
O4	8c	1	0.6318(6)	-0.0766(5)	0.0303(4)
D1	8c	1	0.5068(6)	0.7562(5)	0.2585(9)

Table S15. Anisotropic displacement parameters on Rb_3WO_4D determined from neutron diffraction data at room temperature

Anisotropic displacement parameters [Å²]						
**Atom**	**U_{11}**	**U_{22}**	**U_{33}**	**U_{12}**	**U_{13}**	**U_{23}**
W1	0.0181(20)	0.016(2)	0.030(3)	-0.008(3)	-0.011(3)	-0.004(3)
Rb1	0.0243(18)	0.035(3)	0.037(3)	0.003(2)	-0.018(3)	0.0047(19)
Rb2	0.029(4)	0.027(2)	0.028(2)	0.003(2)	-0.0096(20)	-0.002(2)
Rb3	0.032(4)	0.017(2)	0.041(3)	0.004(3)	0.007(2)	0.008(2)
O1	0.016(4)	0.064(4)	0.055(4)	0.008(3)	-0.021(3)	0.006(3)
O2	0.065(5)	0.053(4)	0.015(3)	-0.022(3)	0.013(3)	-0.013(2)
O3	0.071(5)	0.032(3)	0.044(3)	0.026(3)	-0.024(3)	0.004(3)
O4	0.047(4)	0.051(4)	0.038(4)	-0.021(4)	0.022(3)	-0.012(3)
D1	0.0542(19)	0.042(3)	0.077(4)	0.023(3)	0.007(3)	-0.008(4)

Table S16. Selected interatomic distances in Rb_3WO_4D determined from neutron diffraction data

**Atom 1**	**Atom 2**	**Distance (Å)**
W1	O4	1.7347(97)
	O1	1.7742(93)
	O2	1.7832(68)
	O3	1.7839(66)
	Ø bond length	1.769 Å
Rb1	D1	2.9652(86)
		3.0025(86)
Rb2	D1	2.8529(117)
		2.9230(117)
Rb3	D1	2.8440(73)
		2.9046(74)

Table S17. Tetrahedron angles of the tungstate ions in Rb_3WO_4D determined from neutron diffraction data

**Atom 1**	**Atom 2**	**Atom 3**	**Angle 2-1-3 (°)**
W1	O4	O1	111.59(50)
	O4	O2	112.17(37)
	O4	O3	111.26(35)
	O1	O2	108.43(35)
	O1	O3	107.96(37)
	O2	O3	105.11(35)
		Ø tetrahedron angle	109.42°

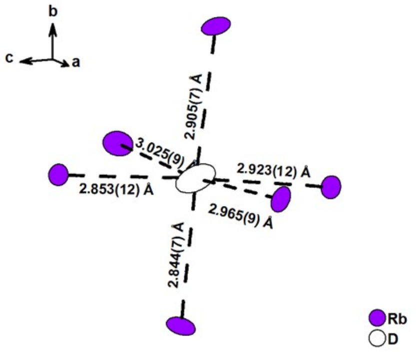

Figure S5 Bond lengths and depiction of the distorted Rb_6D-octahedron

2.1.2 Supplementary X-ray diffraction data (Hydridic samples)

Rb_3MoO_4H

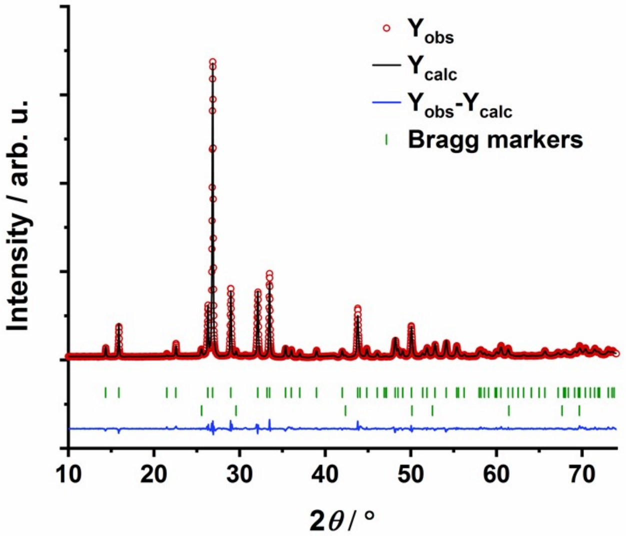

Figure S6. Rietveld refinement plot of Rb_3MoO_4H obtained from X-ray diffraction data at room temperature with the program Fullprof[1]. Bragg markers: Rb_3MoO_4H (top) (97.9(10) wt.%); RbH (bottom) 2.02(2) wt.%). R_p = 5.90%, R_{wp} = 8.49%, R_{exp} = 3.63%, R_{bragg} = 4.01%, χ^2 = 5.90

Table S18. Crystallographic data on Rb_3MoO_4H determined from X-ray diffraction data at room temperature

**Cell parameters**<br>a = 7.8741(4) Å, c = 12.3172(7) Å;<br>a/b=1.00, b/c= 0.6393, V = 760.68(7) Å^3						
**Atom**	**Wyckoff position**	**Site**	**x/a**	**y/b**	**z/c**	**B_{iso} (Å^2)**
Mo	4b	-42*m*	0	½	¼	0.0013(13)
Rb1	8h	*m*2*m*	0.18962(15)	x+½	0	0.0036(12)
Rb2	4a	422	0	0	¼	0.0129(15)
O1	16l	*m*	0.1232(6)	x+½	0.6698(6)	0.005(3)
H1	4c	4/*m*	0	0	0	0.0507*

*The isotropic displacement factor of hydrogen has been set to a realistic value, as no refinement thereof with X-ray diffraction data was possible.

Table S19. Selected interatomic distances and tetrahedron angles in Rb_3MoO_4H determined from X-ray diffraction data

**Atom 1**	**Atom 2**	**Distance (Å)**	
Mo1	O1	1.6906(58)	
Rb1	H1	2.8639(12)	
Rb2		3.0793(2)	
Tetrahedron angle			
**Atom 1**	**Atom 2**	**Atom 3**	**Angle 2-1-3 (°)**
Mo1	O1	O1	108.489(235)
	O1	O1	109.965(265)
		Ø tetrahedron angle	109.227

Cs_3MoO_4H

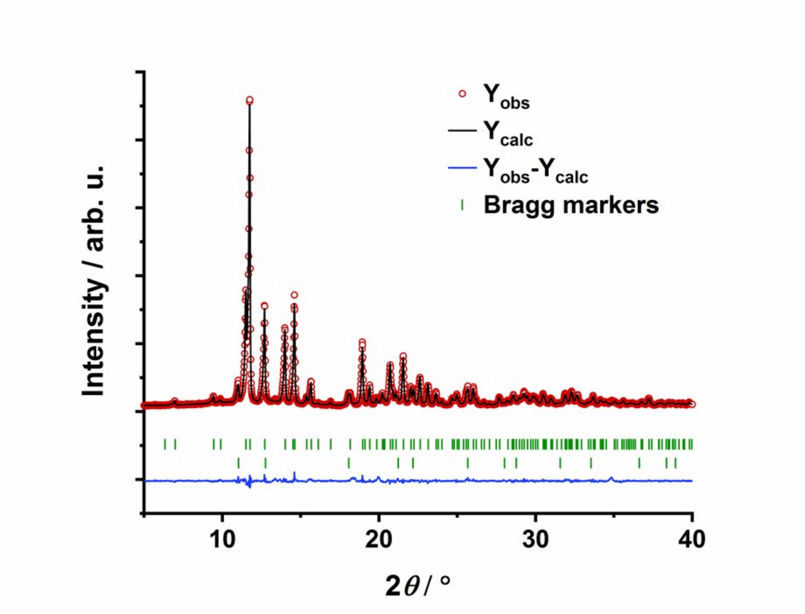

Figure S7. Rietveld refinement plot of Cs_3MoO_4H obtained from X-ray diffraction data at room temperature with the program Fullprof[1]. Bragg markers: Cs_3MoO_4H (top) (94.3(10) wt.%); CsH (bottom) 5.7(2) wt.%). R_p = 3.88%, R_{wp} = 5.40%, R_{exp} = 3.32%, R_{bragg} = 2.93%, χ^2 = 2.65

Table S20. Crystallographic data on Cs_3MoO_4H determined from X-ray diffraction data at room temperature

**Cell parameters**<br>a = 8.2232(2)Å, c = 12.8149(3)Å;<br>a/b=1.00, b/c= 0.6417, V = 866.55(3) Å^3						
**Atom**	**Wyckoff position**	**Site**	**x/a**	**y/b**	**z/c**	**B$_{iso}$ (Å^2)**
Mo	4b	*-42m*	0	½	¼	0.0156(11)
Cs1	8h	*m2m*	0.18685(13)	x+½	0	0.0203(8)
Cs2	4a	422	0	0	¼	0.0284(10)
O1	16l	*m*	0.1246(9)	x+½	0.6736(8)	0.034(4)
H1	4c	*4/m*	0	0	0	0.0507*

*The isotropic displacement factor of hydrogen has been set to a realistic value, as no refinement thereof with X-ray diffraction data was possible.

Table S21. Selected interatomic distances and tetrahedron angles in Rb_3MoO_4H determined from X-ray diffraction data

**Atom 1**	**Atom 2**	**Distance (Å)**	
Mo1	O1	1.7488(84)	
Cs1	H1	2.9986(11)	
Cs2		3.1236(90)	
Tetrahedron angle			
**Atom 1**	**Atom 2**	**Atom 3**	**Angle 2-1-3 (°)**
Mo1	O1	O1	111.908(350)
	O1	O1	108.267(383)
		Ø tetrahedron angle	110.088

Cs_3WO_4H

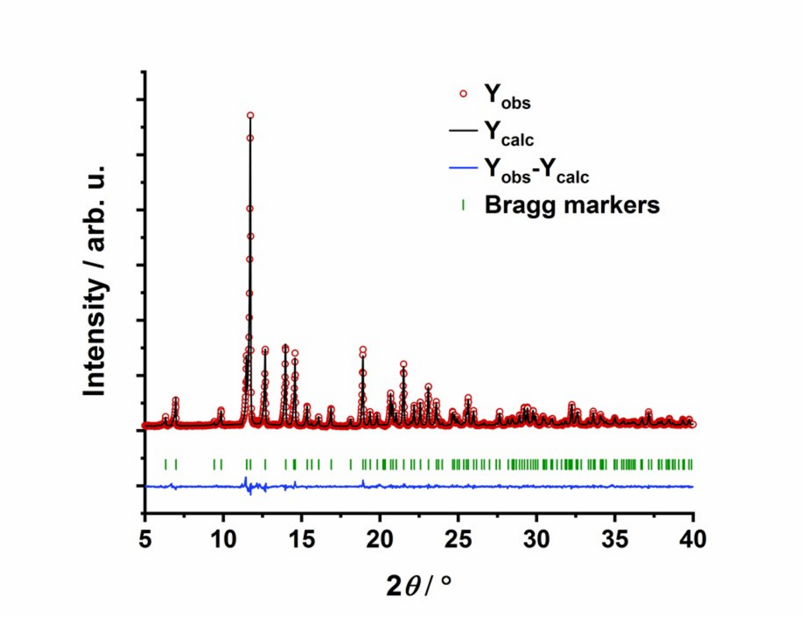

Figure S8. Rietveld refinement plot of Cs_3WO_4H obtained from X-ray diffraction data at room temperature with the program Fullprof[1]. Bragg markers: Cs_3WO_4H. R_p = 4.83%, R_{wp} = 6.65%, R_{exp} = 5.38%, R_{bragg} = 3.83%, χ^2 = 1.53

Table S22. Crystallographic data on Cs_3WO_4H determined from X-ray diffraction data at room temperature

Cell parameters
a = 8.2391(2) Å, c = 12.8405(3) Å;
a/b=1.00, b/c= 0.6417, V = 871.66(3) Å^3

Atom	Wyckoff position	Site	x/a	y/b	z/c	B_{iso} (Å^2)
W	4b	*-42m*	0	½	¼	0.0049(8)
Cs1	8h	*m2m*	0.18890(14)	x+½	0	0.0115(8)
Cs2	4a	422	0	0	¼	0.0177(10)
O1	16l	*m*	0.1251(10)	x+½	0.6668(9)	0.029(5)
H1	4c	4/*m*	0	0	0	0.0507*

*The isotropic displacement factor of hydrogen has been set to a realistic value, as no refinement thereof with X-ray diffraction data was possible.

Table S23. Selected interatomic distances and tetrahedron angles in Cs_3WO_4H determined from X-ray diffraction data

Atom 1	Atom 2	Distance (Å)
W1	O1	1.8072(95)
Cs1	H1	2.9987(12)
Cs2		3.2101(1)

Tetrahedron angle

Atom 1	Atom 2	Atom 3	Angle 2-1-3 (°)
W1	O1	O1	110.454(421)
	O1	O1	107.524(382)
		Ø tetrahedron angle	108.989

Rb_3WO_4H

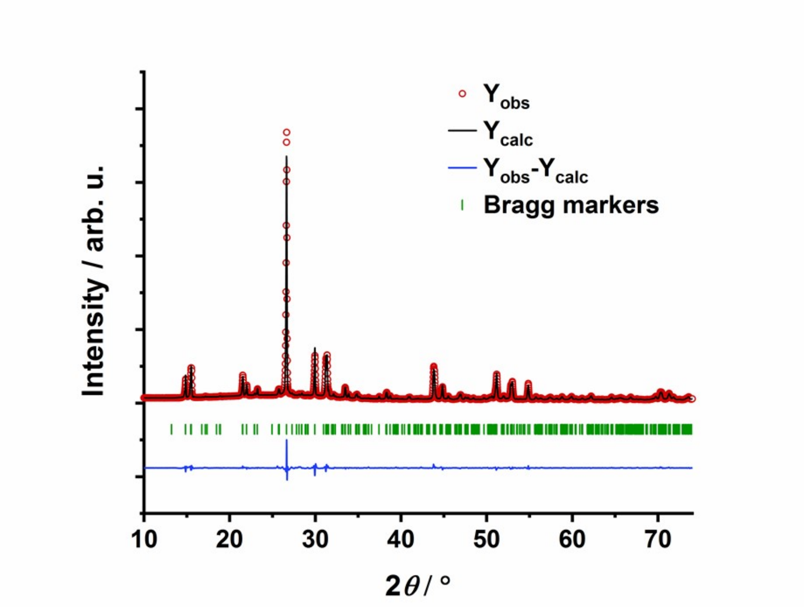

Figure S9 Rietveld refinement plot of Rb_3WO_4H at room temperature based on X-ray diffraction data. Bragg markers Rb_3WO_4H. R_p = 2.91%, R_{wp} = 4.38%, R_{exp} = 1.03%, R_{bragg} = 2.78%, χ^2 = 17.9. Due to very high scattering intensity, necessary to properly resolve the splitting of the reflections, the data quality is very high. R_{exp} is therefore estimated to be very low, resulting in a rather high goodness of fit as $\chi^2 = R_{wp}^2 / R_{exp}^2$.

Table S24. Crystallographic data on Rb_3WO_4H determined from neutron diffraction data at room temperature

Cell parameters
a = 11.9295(3) Å, b = 11.4019(3) Å, c = 11.4516(3) Å; a/b=1.0463 b/c=0.9957 c/a=0.9599
V= 1557.63(7)Å^3

Atom	Wyckoff position	Site	x/a	y/b	z/c	B_{iso} (Å^2)
W1	8c	1	0.7502(4)	0.0065(4)	-0.0164(2)	0.0042(8)
Rb1	8c	1	0.7452(13)	0.2331(6)	0.7296(4)	0.0178(17)
Rb2	8c	1	-0.0008(10)	0.7819(3)	0.0057(11)	0.0172(20)
Rb3	8c	1	0.0007(8)	-0.0078(6)	0.7199(3)	0.0110(19)
O1	8c	1	0.858(2)	-0.022(3)	0.0919(20)	0.036(10)
O2	8c	1	0.787(3)	-0.013(3)	0.840(2)	0.007(10)
O3	8c	1	0.710(3)	0.152(2)	-0.011(3)	0.043(14)
O4	8c	1	0.631(3)	-0.068(2)	0.047(2)	0.026(10)
H1	8c	1	0.50680*	0.75620*	0.25850*	0.0507*

*The atomic position of hydrogen was not determinable via X-ray diffraction and assumed to be the position determined by neutron diffraction. The isotropic displacement factor of hydrogen has been set to a realistic value, as no refinement thereof with X-ray diffraction data was possible.

Table S25. Selected interatomic distances in Rb_3WO_4D determined from neutron diffraction data

Atom 1	Atom 2	Distance (Å)
W1	O4	1.8085(318)
	O1	1.8159(241)
	O2	1.7165(243)
	O3	1.7280(245)
	Ø bond length	1.7672 Å
Rb1	H1	2.9795(154)
		3.00118(155)
Rb2	H1	2.7177(125)
		3.0579(125)
Rb3	H1	2.8439(68)
		2.9040(68)

Table S26. Tetrahedron angles of the tungstate ions in Rb_3WO_4D determined from neutron diffraction data

Atom 1	Atom 2	Atom 3	Angle 2-1-3 (°)
W1	O4	O1	101.44(124)
	O4	O2	121.66(123)
	O4	O3	102.61(120)
	O1	O2	116.74(110)
	O1	O3	110.12(143)
	O2	O3	103.28(155)
		Ø tetrahedron angle	109.31°

2.2 Ionic radii for determination of the Goldschmidt tolerance factors

The ionic radii of the complex oxoanions are taken from the determined bond lengths of the *M*-O bond (as depicted above) plus the ionic radius of oxygen in sixfold coordination sphere. Ionic radii are taken from Shannon in respect of the coordination spheres of the ions.[12]

The hydride ion is known to have high polarizability and therefore shows different ionic radii, depending on the chemical environment. We have set the ionic radius of the hydride anion to be 1.399 Å as suggested from Shannon and also from Lang and Smith[12,13]. This approach is expected to return consistent set of tolerance factors that enable a discussion of chemical packing as a factor for differences in their crystal chemistries.

Table S27. Ionic radii for determination of the Goldschmidt tolerance factors

Ionic species	Ionic radius	Coordination
Na^+	1.02	Sixfold
K^+	1.38	Eightfold/tenfold
Rb^+	1.52	Sixfold (Rb_3WO_4H)
Rb^+	1.63	Eightfold/tenfold
Cs^+	1.67	Eightfold/tenfold
MoO_4^{2-}	3.170	-
WO_4^{2-}	3.180	-
SO_4^{2-}	2.87	-
H^-	1.399	Sixfold
F^-	1.33	Sixfold
O^{2-}	1.40	Sixfold

2.3 Crystal structure of the K_3SO_4F-structure type

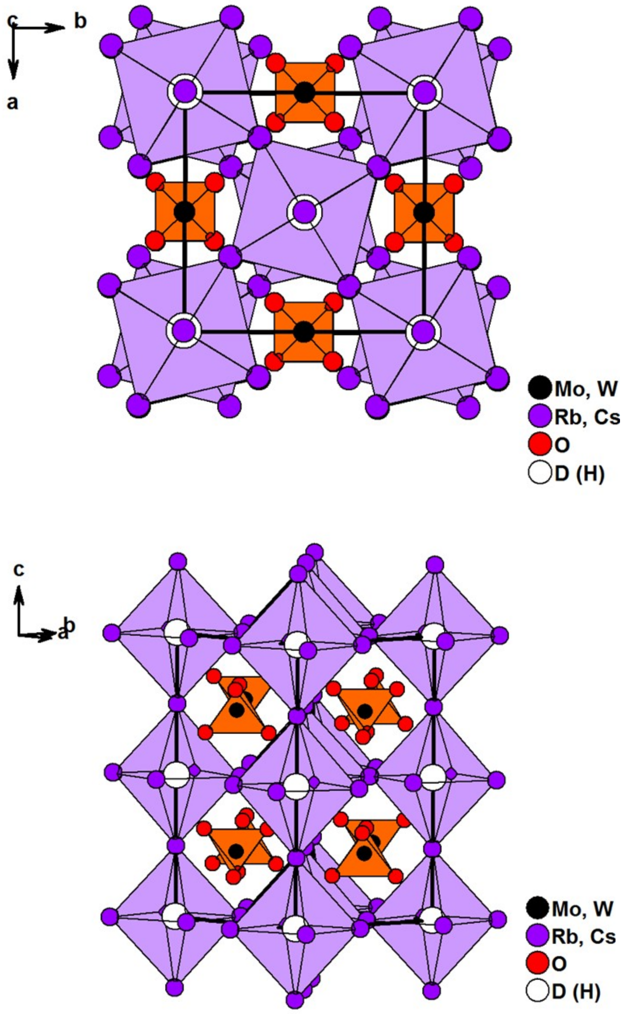

Figure S10. Crystal structure of the tetragonal phases along the c-axis (top) and along the a/b plane (bottom). The Rb_6D /Cs_6D octahedrons are depicted lilac, the tungstate ions are depicted as orange tetrahedrons.

2.4 Difference Fourier maps

Rb_3MoO_4D

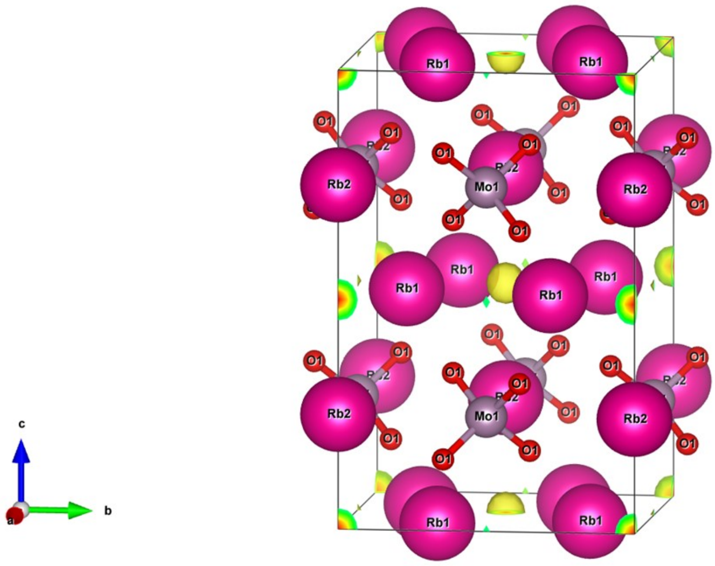

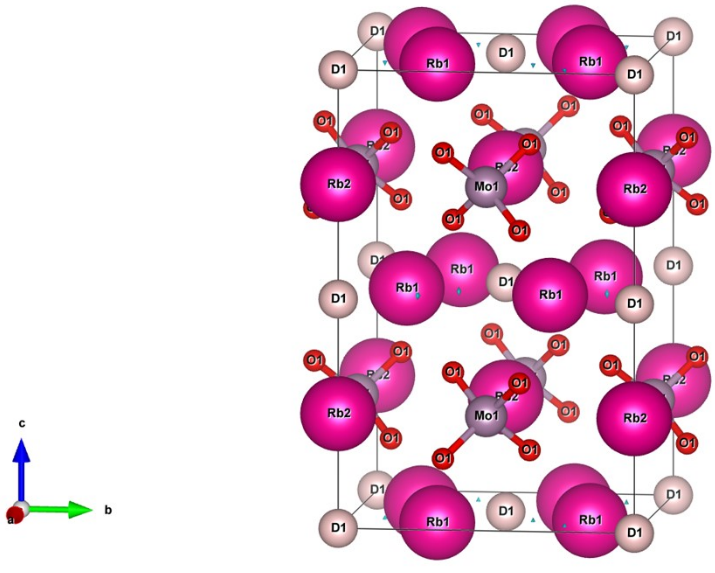

Figure S11. Difference Fourier map of the refinement Rb_3MoO_4□ (top) with an unoccupied deuterium position (symbol □, in FullProf the occupation and B_{iso} were set to 0). Rubidium is shown in pink, tungsten in grey and oxygen in red. The positive, residual density (yellow isosurface, projected at an isosurface level of 0.090) agrees with the experimentally determined deuterium position in Rb_3MoO_4D. For comparison: difference Fourier map of the refinement Rb_3MoO_4D (bot). Here, no residual density is found at a ca. twenty times lower isosurface level of 0.0058. The lower the isosurface leve is set, the more sensitively residual neutron density is shown.
In addition, no residual neutron density is found next to the now occupied deuteride position. This excludes a likely hydroxide/deuteroxide species as no neutron density of a closely bound atom is observable. Graphic representation is shown in VESTA[14].

Cs_3MoO_4D

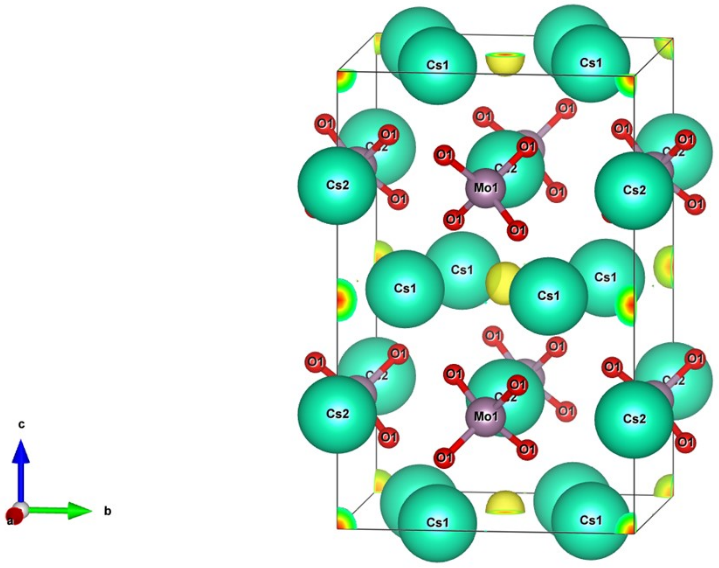

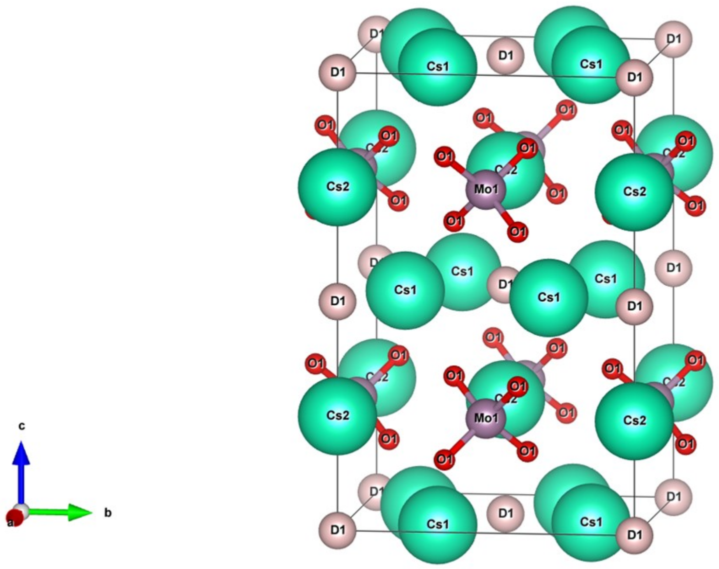

Figure S12. Difference Fourier map of the refinement $Cs_3MoO_4□$ (top) with an unoccupied deuterium position (symbol □, in FullProf the occupation and B_{iso} were set to 0). Rubidium is shown in pink, tungsten in grey and oxygen in red. The positive, residual density (yellow isosurface, projected at an isosurface level of 0.074) agrees with the experimentally determined deuterium position in Cs_3MoO_4D. For comparison: difference Fourier map of the refinement Cs_3MoO_4D (bot). Here, no residual density is found at a ten times lower isosurface level of 0.0075. The lower the isosurface leve is set, the more sensitively residual neutron density is shown. Again, no residual neutron density is found next to the here occupied deuteride position. This excludes a likely hydroxide/deuteroxide species as no neutron density of a closely bound atom is observable. Graphic representation is shown in VESTA[14].

Cs_3WO_4D

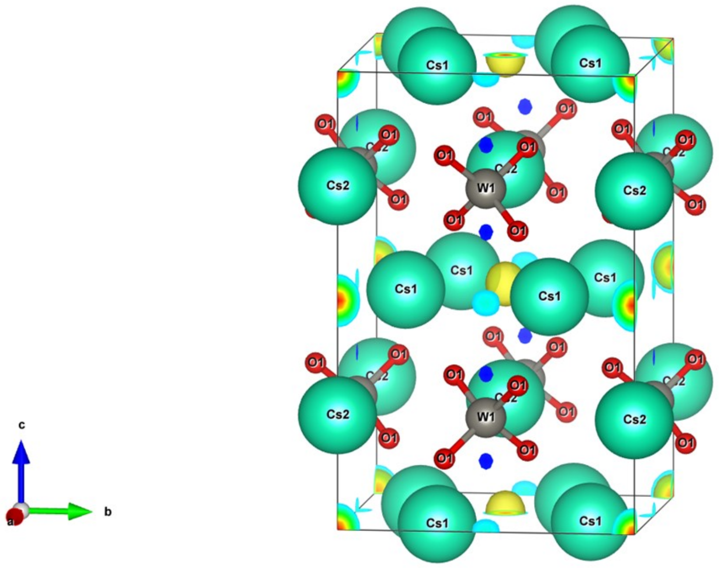

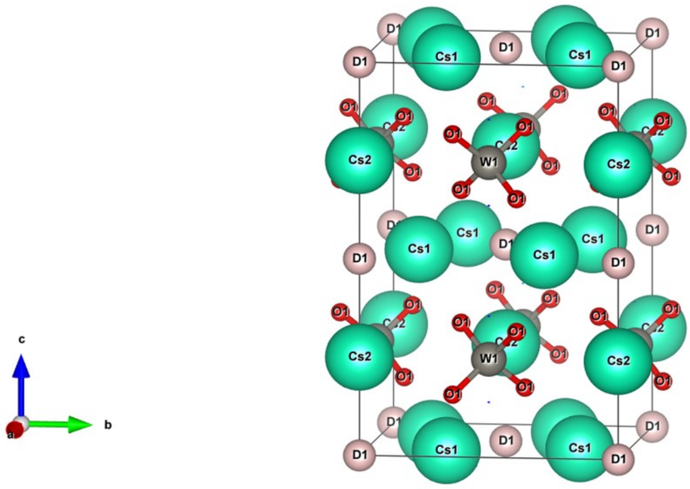

Figure S13. Difference Fourier map of the refinement $Cs_3WO_4□$ (top) with an unoccupied deuterium position (symbol □, in FullProf the occupation and B_{iso} were set to 0). Rubidium is shown in pink, tungsten in grey and oxygen in red. The positive, residual density (yellow isosurface, projected at an isosurface level of 0.043) agrees with the experimentally determined deuterium position in Cs_3WO_4D. For comparison: difference Fourier map of the refinement Cs_3WO_4D (bot). Here, no residual density is found at a five times lower isosurface level of 0.009. The lower the isosurface leve is set, the more sensitively residual neutron density is shown. No residual neutron density is found next to the now occupied deuteride position. This excludes a likely hydroxide/deuteroxide or any other different species as no neutron density of a closely bound atom is observable. Graphic representation is shown in VESTA[14].

Rb_3WO_4D

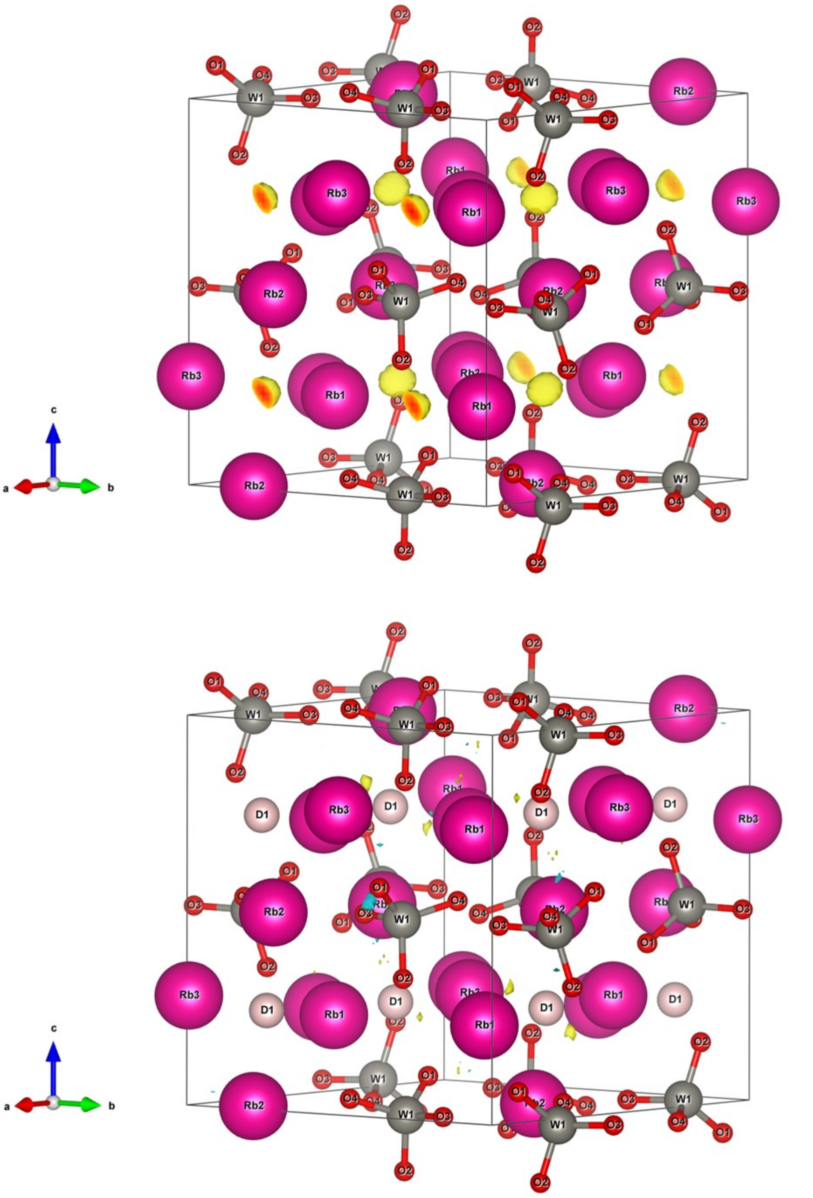

Figure S14. Difference Fourier map of the refinement Rb_3WO_4□ (top) with an unoccupied deuterium position (symbol □, in FullProf the occupation and B_{iso} were set to 0). Rubidium is shown in pink, tungsten in grey and oxygen in red. The positive, residual density (yellow isosurface, projected at an isosurface level of 0.055) agrees with the experimentally determined deuterium position in Rb_3WO_4D. For comparison: difference Fourier map of the refinement Rb_3WO_4D (bot). Here, no residual density is found at a ten times lower isosurface level of 0.0055. Once again, no residual neutron density is found next to the now occupied deuteride position. This excludes a likely hydroxide/deuteroxide species as no neutron density of a closely bound atom is observable. Graphic representation is shown in VESTA[14].

2.5 ^{2}H MAS NMR Spectroscopy

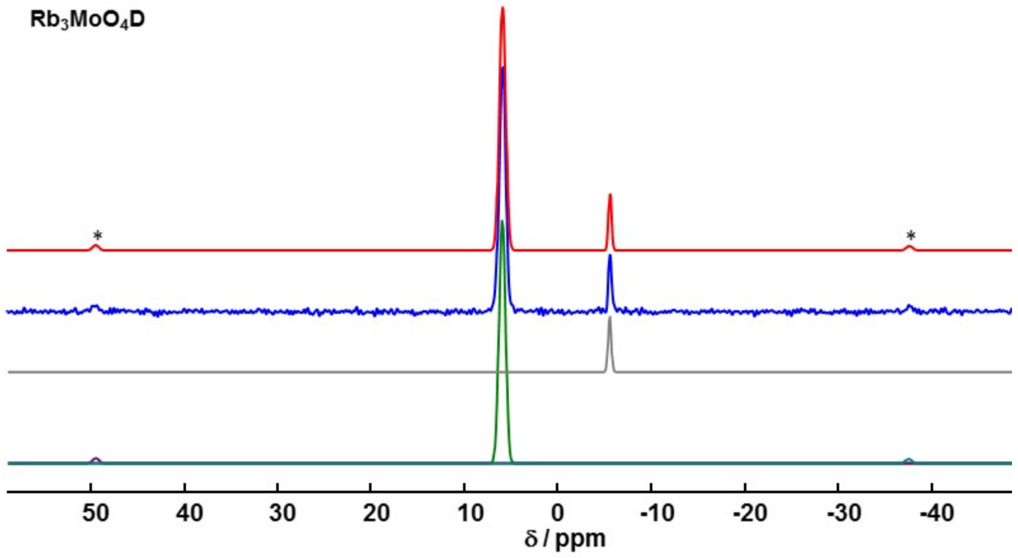

Figure S15. Full ^{2}H MAS spectrum of Rb_3MoO_4D. Rotational sidebands of the target compound signal are masked with asterisks. Red line full fit, blue line experimental spectrum. Lines below depict the fits of the individual signals.

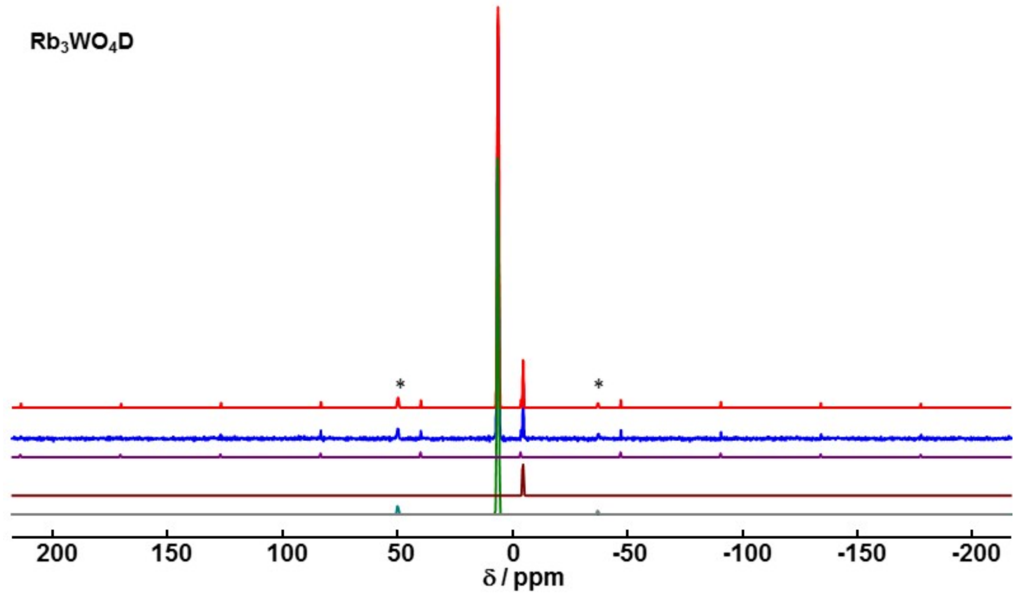

Figure S16. Full ^{2}H MAS spectrum of Rb_3WO_4D. Rotational sidebands of the target compound signal are masked with asterisks. Red line full fit, blue line experimental spectrum. Lines below depict the fits of the individual signals.

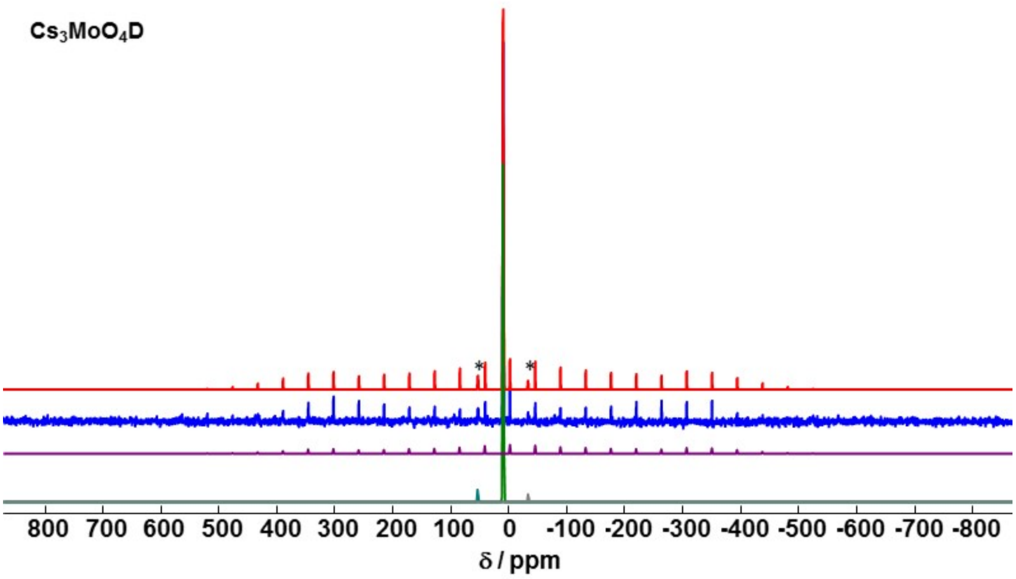

Figure S17. Full ^{2}H MAS spectrum of Cs_3MoO_4D. Rotational sidebands of the target compound signal are masked with asterisks. Red line full fit, blue line experimental spectrum. Lines below depict the fits of the individual signals.

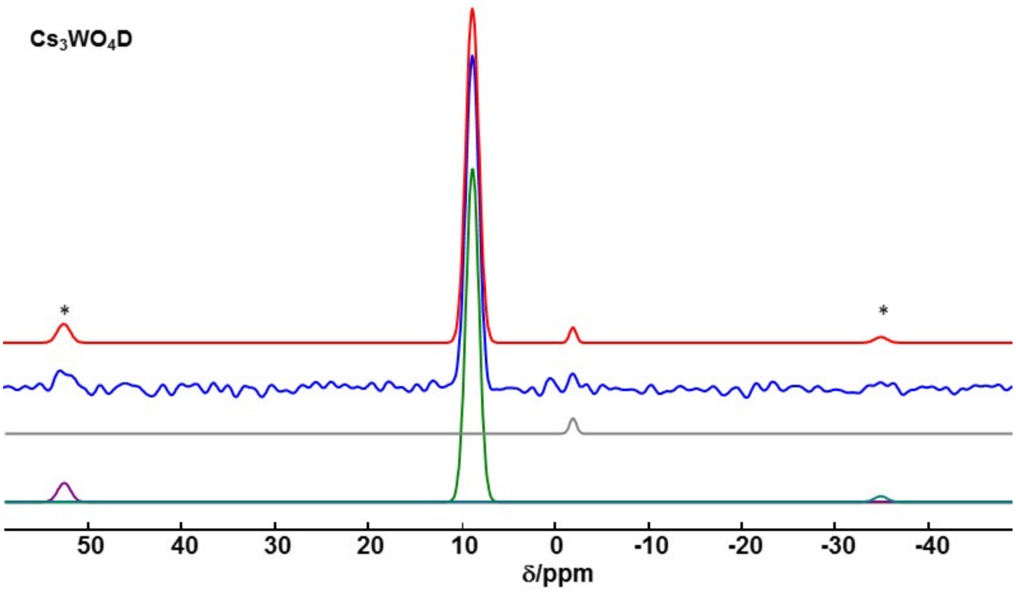

Figure S18. Full ^{2}H MAS spectrum of Cs_3WO_4D. Rotational sidebands of the target compound signal are masked with asterisks. Red line full fit, blue line experimental spectrum. Lines below depict the fits of the individual signals.

2.6 Calculated electronic band structures and UV/Vis Tauc plots

Rb_3MoO_4H

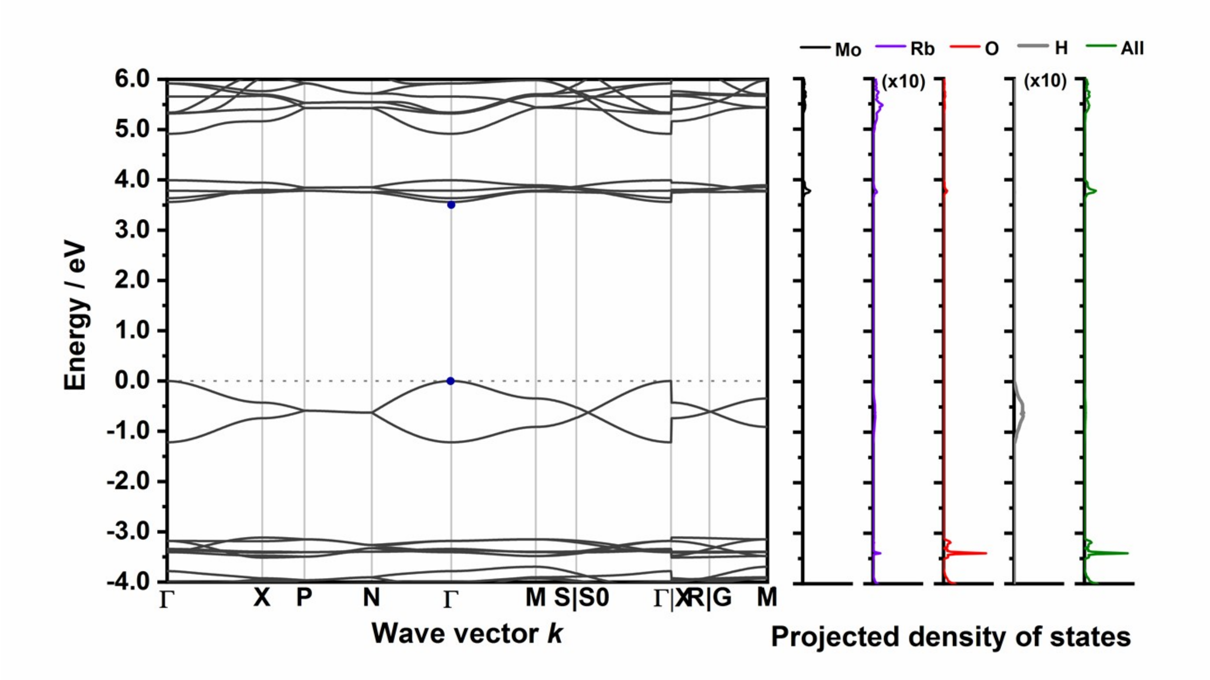

Figure S19. Calculated electronic band structure of Rb_3MoO_4H and projected density of states (DFT-PBE0). The blue dots indicate the direct band gap character. The DOS of Rubidium and Hydrogen are enhanced for better visibility. The band paths in the reciprocal space have been determined by the Seek-path webservice[15–17].

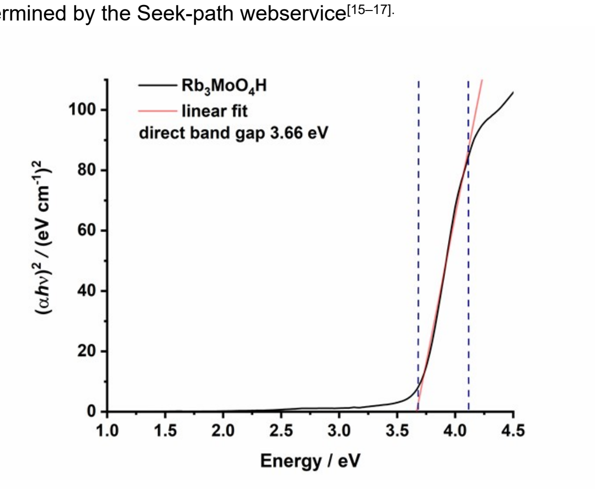

Figure S20. Tauc-plots of Rb_3MoO_4H determined from UV/VIS absorption spectroscopy. For the determination of a direct transition character, the coefficient r (see experimental details) has been set to r = ½, direct transition (left side). The blue vertical dashed lines indicate the area for the linear fit. The resulting determined direct band gap of 3.66 eV is matching the predicted band gap of 3.56 eV.

Cs_3MoO_4H

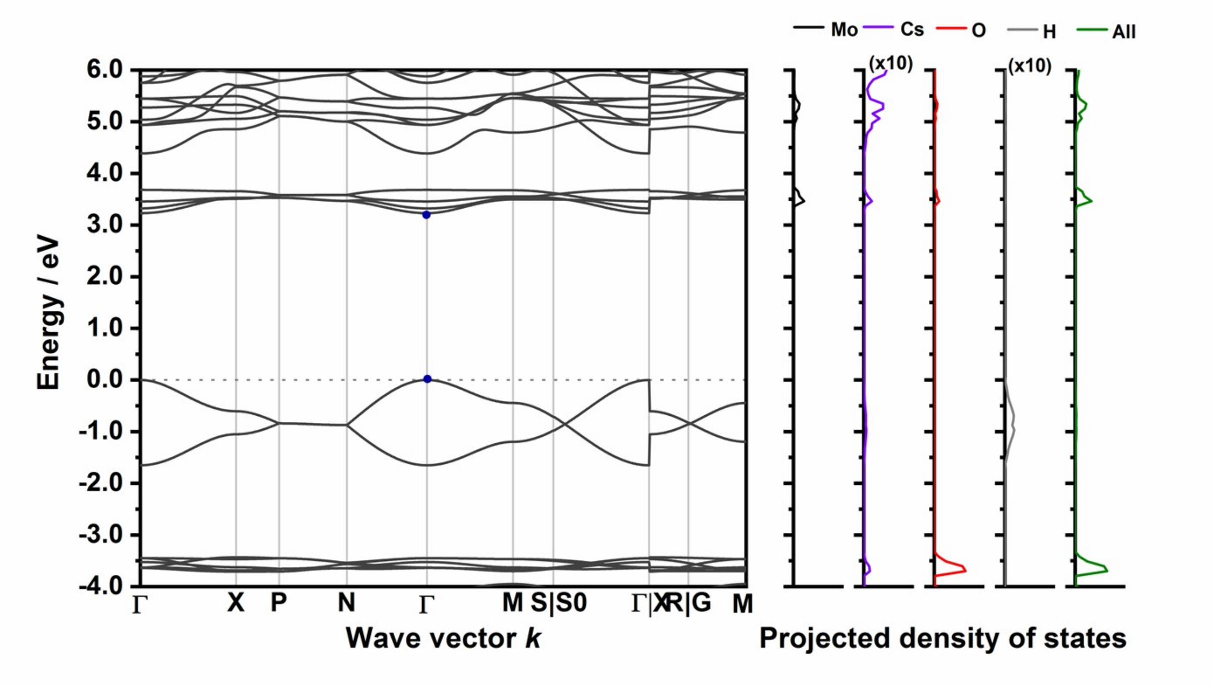

Figure S21. Calculated electronic band structure of Cs_3MoO_4H and projected density of states (DFT-PBE0). The blue dots indicate the direct band gap character. The DOS of Cesium and Hydrogen are enhanced for better visibility. The band paths in the reciprocal space have been determined by the Seek-path webservice[15–17].

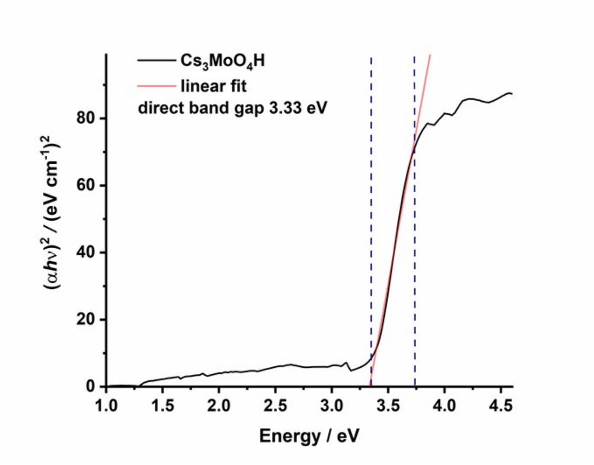

Figure S22. Tauc-plots of Cs_3MoO_4H determined from UV/VIS absorption spectroscopy. For the determination of a direct or indirect transition character, the coefficient r (see experimental details) has been set to r = ½. The blue vertical dashed lines indicate the area for the linear fit. The resulting determined direct band gap of 3.33 eV is matching the predicted band gap of 3.23 eV.

Cs_3WO_4H

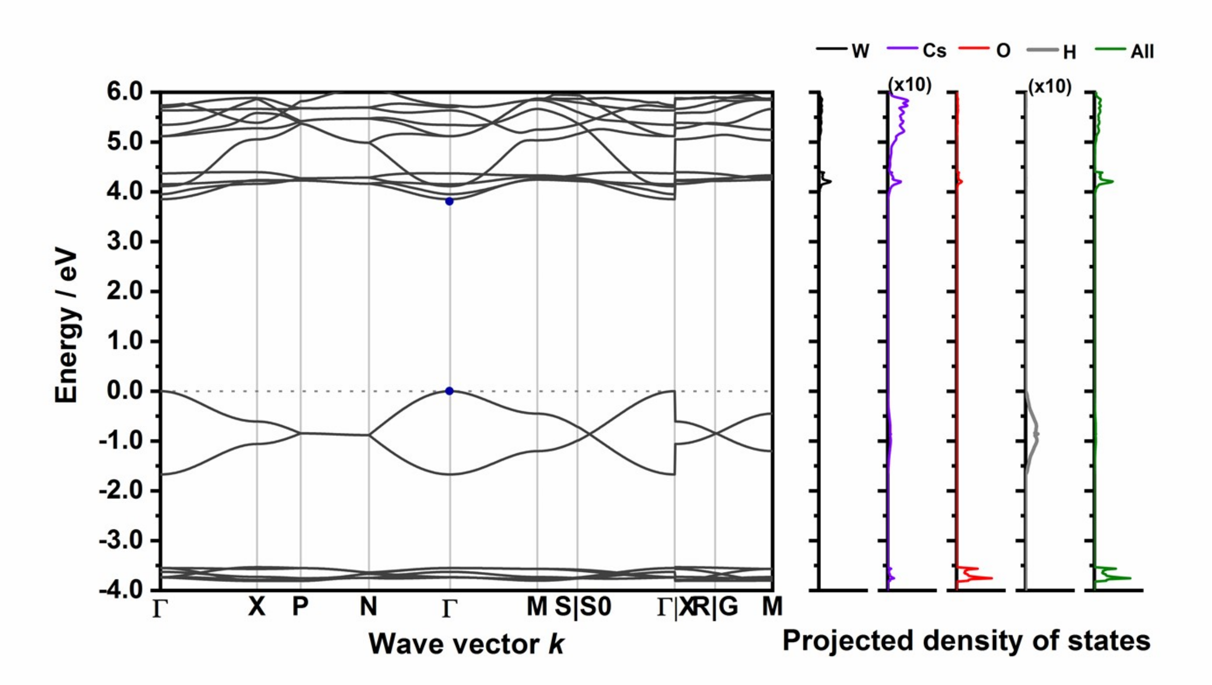

Figure S23. Calculated electronic band structure of Cs_3WO_4H and projected density of states (DFT-PBE0). The blue dots indicate the direct band gap character. The DOS of Rubidium and Hydrogen are enhanced for better visibility. The band paths in the reciprocal space have been determined by the Seek-path webservice[15–17].

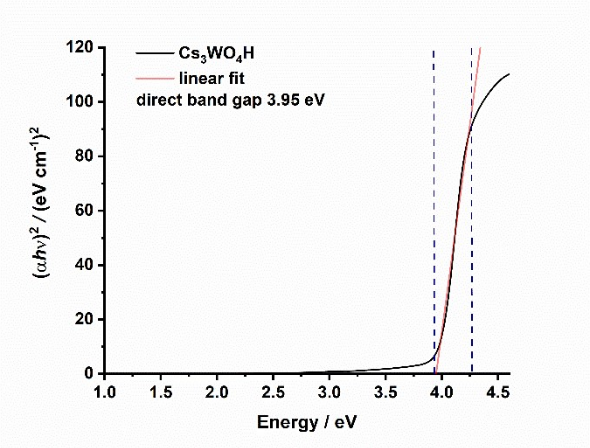

Figure S24. Tauc-plots of Cs_3WO_4H determined from UV/VIS absorption spectroscopy. For the determination of a direct or indirect transition character, the coefficient r (see experimental details) has been set to r = ½. The blue vertical dashed lines indicate the area for the linear fit. The resulting determined direct band gap of 3.95 eV is matching the predicted band gap of 3.85 eV.

2.7 Vibrational Spectroscopy (experimental and DFT-PBE0)

Rb_3MoO_4H

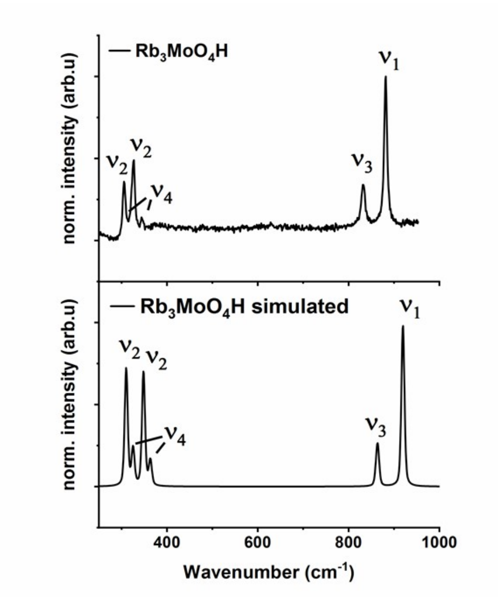

Figure S25. Experimental Raman spectrum of Rb_3MoO_4H (top) and simulated Raman spectrum of Rb_3MoO_4H (bottom).

Table S28.Vibrational Raman data of Rb_3MoO_4H obtained by quantum chemical calculations.

**Simulated Raman data on Rb_3MoO_4H**			
**Frequency (cm^{-1})**	**Γ_{irrep}**	**Intensity (arbitrary units)**	**Assignment**
46	E_g	33	Lattice vibrations
56	E_g	31	Lattice vibrations
69	A_{1g}	56	Lattice vibrations
72	B_{1g}	34	Lattice vibrations
76	B_{2g}	2	Lattice vibrations
95	E_g	188	MoO_4^{2-} antisymmetric bending coupled with lattice vibrations
97	E_g	1	Lattice vibrations
114	B_{2g}	49	Lattice vibrations
310	B_{1g}	726	MoO_4^{2-} symmetric bending (v_2)
325	E_g	225	MoO_4^{2-} antisymmetric bending (v_4)
348	A_{1g}	705	MoO_4^{2-} symmetric bending (v_2)
363	B_{2g}	154	MoO_4^{2-} antisymmetric bending (v_4)
861	B_{2g}	69	MoO_4^{2-} antisymmetric stretching (v_3)
864	E_g	212	MoO_4^{2-} antisymmetric stretching (v_3)
919	A_{1g}	1000	MoO_4^{2-} symmetric stretching (v_1)

Cs_3MoO_4H

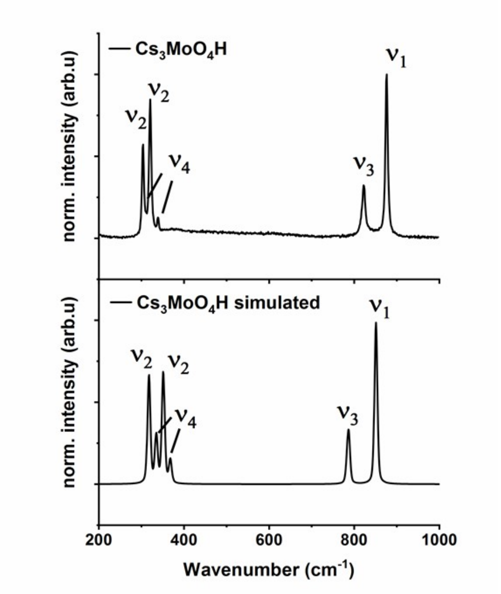

Figure S26. Experimental Raman spectrum of Cs_3MoO_4H (top) and simulated Raman spectrum of Cs_3MoO_4H (bottom).

Table S29.Vibrational Raman data of Cs_3MoO_4H obtained by quantum chemical calculations.

**Simulated Raman data on Cs_3MoO_4H**			
**Frequency (cm^{-1})**	**Γ_{irrep}**	**Intensity (arbitrary units)**	**Assignment**
32	E_g	1	Lattice vibrations
39	E_g	83	Lattice vibrations
50	A_{1g}	137	Lattice vibrations
56	B_{2g}	10	Lattice vibrations
56	B_{1g}	168	Lattice vibrations
80	E_g	39	Lattice vibrations
101	B_{2g}	46	Lattice vibrations
141	E_g	102	MoO_4^{2-} antisymmetric bending coupled with lattice vibrations
318	B_{1g}	670	MoO_4^{2-} symmetric bending (v_2)
334	E_g	289	MoO_4^{2-} antisymmetric bending (v_4)
351	A_{1g}	685	MoO_4^{2-} symmetric bending (v_2)
367	B_{2g}	142	MoO_4^{2-} antisymmetric bending (v_4)
783	B_{2g}	58	MoO_4^{2-} antisymmetric stretching (v_3)
786	E_g	306	MoO_4^{2-} antisymmetric stretching (v_3)
850	A_{1g}	1000	MoO_4^{2-} symmetric stretching (v_1)

Cs_3WO_4H

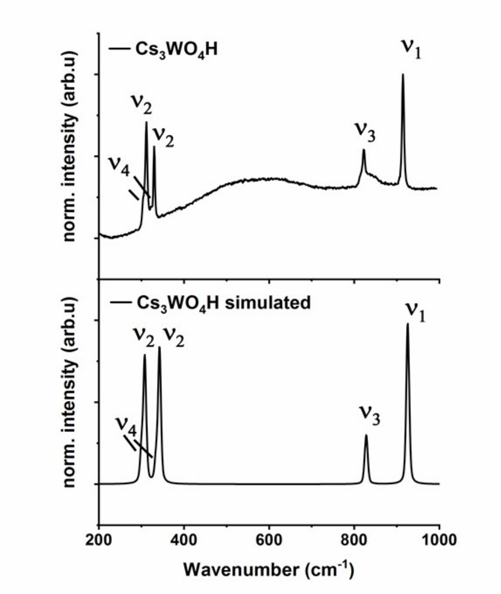

Figure S27. Experimental Raman spectrum of Cs_3WO_4H (top) and simulated Raman spectrum of Cs_3WO_4H (bottom).

Table S30.Vibrational Raman data of Cs_3MoO_4H obtained by quantum chemical calculations.

**Simulated Raman data on Cs_3WO_4H**			
**Frequency (cm^{-1})**	**Γ_{irrep}**	**Intensity (arbitrary units)**	**Assignment**
25	E_g	0	Lattice vibrations
36	E_g	55	Lattice vibrations
47	A_{1g}	214	Lattice vibrations
50	B_{2g}	5	Lattice vibrations
55	B_{1g}	288	Lattice vibrations
70	E_g	60	Lattice vibrations
84	B_{2g}	76	Lattice vibrations
86	E_g	277	WO_4^{2-} antisymmetric bending coupled with lattice vibrations
300	E_g	210	WO_4^{2-} antisymmetric bending (v_4)
308	B_{1g}	766	WO_4^{2-} symmetric bending (v_2)
335	B_{2g}	147	WO_4^{2-} antisymmetric bending (v_4)
343	A_{1g}	833	WO_4^{2-} symmetric bending (v_2)
826	B_{2g}	47	WO_4^{2-} antisymmetric stretching (v_3)
828	E_g	266	WO_4^{2-} antisymmetric stretching (v_3)
926	A_{1g}	1000	WO_4^{2-} symmetric stretching (v_1)

The Raman spectrum of Cs_3WO_4H shows a broad background within the spectrum due to self-fluorescence of the sample.

Rb_3WO_4H

Table S31.Vibrational Raman data of Rb_3WO_4H obtained by quantum chemical calculations.

Simulated Raman data on Rb_3WO_4H			
**Frequency (cm^{-1})**	**Γ_{irrep}**	**Intensity (arbitrary units)**	**Assignment**
26 - 146	-	> 20	Lattice vibrations
307	B_{3g}	14	WO_4^{2-} antisymmetric bending (v_4)
309	B_{1g}	31	WO_4^{2-} antisymmetric bending (v_4)
311	A_g	15	WO_4^{2-} antisymmetric bending (v_4)
313	B_{2g}	30	WO_4^{2-} antisymmetric bending (v_4)
314	B_{3g}	25	WO_4^{2-} antisymmetric bending (v_4)
315	B_{1g}	101	WO_4^{2-} antisymmetric bending (v_4)
316	A_g	11	WO_4^{2-} antisymmetric bending (v_4)
317	B_{2g}	7	WO_4^{2-} antisymmetric bending (v_4)
320	B_{2g}	28	WO_4^{2-} antisymmetric bending (v_4)
323	B_{2g}	485	WO_4^{2-} symmetric bending (v_2)
323	B_{3g}	5	WO_4^{2-} symmetric bending (v_2)
324	A_g	67	WO_4^{2-} symmetric bending (v_2)
324	B_{3g}	22	WO_4^{2-} symmetric bending (v_2)
325	B_{1g}	9	WO_4^{2-} antisymmetric bending (v_4)
330	B_{3g}	212	WO_4^{2-} symmetric bending (v_2)
333	B_{1g}	188	WO_4^{2-} symmetric bending (v_2)
334	A_g	198	WO_4^{2-} symmetric bending (v_2)
338	B_{2g}	23	WO_4^{2-} symmetric bending (v_2)
340	A_g	210	WO_4^{2-} symmetric bending (v_2)
347	B_{1g}	25	WO_4^{2-} symmetric bending (v_2)
585	A_g	4	Parallel vertical hydride vibrations
588	B_{1g}	24	Parallel vertical hydride vibrations
660	B_{3g}	0	Diagonal hydride vibrations
675	B_{2g}	2	Parallel vertical hydride vibrations
715	A_g	2	Parallel horizontal hydride vibrations
724	B_{3g}	0	Diagonal horizontal hydride vibrations
725	A_g	9	Parallel horizontal hydride vibrations
727	B_{3g}	1	Diagonal horizontal hydride vibrations
770	B_{1g}	1	Antiparallel vertical hydride vibrations
779	B_{2g}	0	Antiparallel horizontal hydride vibrations
804	B_{2g}	1	Hydride symmetric vibrations
810	B_{1g}	15	Hydride symmetric vibrations
821	A_g	121	WO_4^{2-} antisymmetric stretching (v_3)
822	B_{2g}	9	WO_4^{2-} antisymmetric stretching coupled with symmetric hydride vibrations (v_{3H})
825	B_{1g}	7	WO_4^{2-} antisymmetric stretching coupled with symmetric hydride vibrations (v_{3H})
827	A_g	84	WO_4^{2-} antisymmetric stretching (v_3)
837	B_{3g}	34	WO_4^{2-} antisymmetric stretching (v_3)
844	A_g	10	WO_4^{2-} antisymmetric stretching (v_3)
853	B_{3g}	0	WO_4^{2-} antisymmetric stretching coupled with parallel vertical hydride vibrations (v_{3H})
861	B_{1g}	29	WO_4^{2-} antisymmetric stretching coupled with parallel vertical hydride vibrations (v_{3H})
869	B_{2g}	9	WO_4^{2-} antisymmetric stretching coupled with diagonal vertical hydride vibrations (v_{3H})
877	B_{1g}	24	WO_4^{2-} antisymmetric stretching coupled with diagonal vertical hydride vibrations (v_{3H})
879	B_{3g}	61	WO_4^{2-} antisymmetric stretching (v_3)
882	B_{2g}	10	WO_4^{2-} antisymmetric stretching coupled with diagonal vertical hydride vibrations (v_{3H})
930	A_g	1000	WO_4^{2-} symmetric stretching (v_1)
931	B_{2g}	0	WO_4^{2-} symmetric stretching (v_1)
931	B_{1g}	1	WO_4^{2-} symmetric stretching (v_1)
932	B_{3g}	0	WO_4^{2-} symmetric stretching (v_1)

Hydride vibrations along the crystallographic a/b-plane are defined as vertical hydride vibrations whereas vibrations along the a/c-axis are defined as horizontal vibrations.

2.8 Elemental Analysis

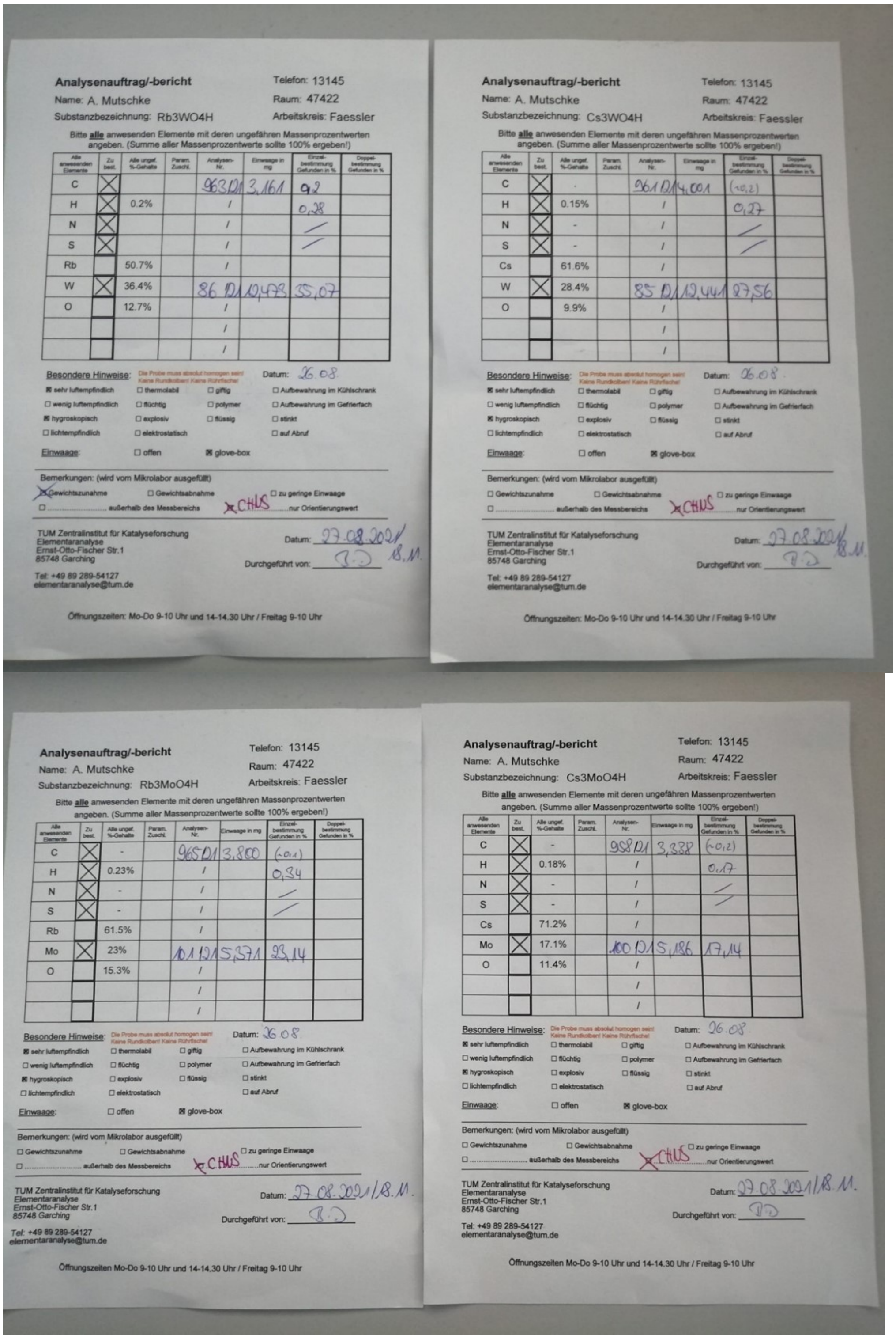

Analysenauftrag/-bericht

Name: A. Mutschke — Telefon: 13145
Raum: 47422
Substanzbezeichnung: Rb3WO4H — Arbeitskreis: Faessler

Bitte **alle** anwesenden Elemente mit deren ungefähren Massenprozentwerten angeben. (Summe aller Massenprozentwerte sollte 100% ergeben!)

Alle anwesenden Elemente	Zu best.	Alle ungef. %-Gehalte	Param. Zuschl.	Analysen-Nr.	Einwaage in mg	Einzel-bestimmung Gefunden in %	Doppel-bestimmung Gefunden in %
C	X			963 D1	3,161	0,2	
H	X	0.2%		/		0,28	
N	X			/		/	
S	X			/		/	
Rb		50.7%		/			
W	X	36.4%		86 D1	12,478	35,07	
O		12.7%		/			
				/			
				/			

Besondere Hinweise: Die Probe muss absolut homogen sein! Keine Rundkolben! Keine Rührfische! — Datum: 26.08

☒ sehr luftempfindlich ☐ thermolabil ☐ giftig ☐ Aufbewahrung im Kühlschrank
☐ wenig luftempfindlich ☐ flüchtig ☐ polymer ☐ Aufbewahrung im Gefrierfach
☒ hygroskopisch ☐ explosiv ☐ flüssig ☐ stinkt
☐ lichtempfindlich ☐ elektrostatisch ☐ auf Abruf

Einwaage: ☐ offen ☒ glove-box

Bemerkungen: (wird vom Mikrolabor ausgefüllt)
☒ Gewichtszunahme ☐ Gewichtsabnahme ☐ zu geringe Einwaage
☐ .... außerhalb des Messbereichs ✗CHNS ....nur Orientierungswert

TUM Zentralinstitut für Katalyseforschung
Elementaranalyse
Ernst-Otto-Fischer Str.1
85748 Garching
Tel: +49 89 289-54127
elementaranalyse@tum.de

Datum: 27.08.2021/ B.M.
Durchgeführt von: B.D.

Öffnungszeiten: Mo-Do 9-10 Uhr und 14-14.30 Uhr / Freitag 9-10 Uhr

Analysenauftrag/-bericht

Name: A. Mutschke — Telefon: 13145
Raum: 47422
Substanzbezeichnung: Cs3WO4H — Arbeitskreis: Faessler

Bitte **alle** anwesenden Elemente mit deren ungefähren Massenprozentwerten angeben. (Summe aller Massenprozentwerte sollte 100% ergeben!)

Alle anwesenden Elemente	Zu best.	Alle ungef. %-Gehalte	Param. Zuschl.	Analysen-Nr.	Einwaage in mg	Einzel-bestimmung Gefunden in %	Doppel-bestimmung Gefunden in %
C	X	-		961 D1	4,001	(~0,2)	
H	X	0.15%		/		0,27	
N	X	-		/		/	
S	X	-		/		/	
Cs		61.6%		/			
W	X	28.4%		85 D1	12,441	27,56	
O		9.9%		/			
				/			
				/			

Besondere Hinweise: Die Probe muss absolut homogen sein! Keine Rundkolben! Keine Rührfische! — Datum: 26.08.

☒ sehr luftempfindlich ☐ thermolabil ☐ giftig ☐ Aufbewahrung im Kühlschrank
☐ wenig luftempfindlich ☐ flüchtig ☐ polymer ☐ Aufbewahrung im Gefrierfach
☒ hygroskopisch ☐ explosiv ☐ flüssig ☐ stinkt
☐ lichtempfindlich ☐ elektrostatisch ☐ auf Abruf

Einwaage: ☐ offen ☒ glove-box

Bemerkungen: (wird vom Mikrolabor ausgefüllt)
☐ Gewichtszunahme ☐ Gewichtsabnahme ☐ zu geringe Einwaage
☐ .... außerhalb des Messbereichs ✗CHNS ....nur Orientierungswert

TUM Zentralinstitut für Katalyseforschung
Elementaranalyse
Ernst-Otto-Fischer Str.1
85748 Garching
Tel: +49 89 289-54127
elementaranalyse@tum.de

Datum: 27.08.2021/ B.M.
Durchgeführt von: B.D.

Öffnungszeiten: Mo-Do 9-10 Uhr und 14-14.30 Uhr / Freitag 9-10 Uhr

Analysenauftrag/-bericht

Name: A. Mutschke — Telefon: 13145
Raum: 47422
Substanzbezeichnung: Rb3MoO4H — Arbeitskreis: Faessler

Bitte **alle** anwesenden Elemente mit deren ungefähren Massenprozentwerten angeben. (Summe aller Massenprozentwerte sollte 100% ergeben!)

Alle anwesenden Elemente	Zu best.	Alle ungef. %-Gehalte	Param. Zuschl.	Analysen-Nr.	Einwaage in mg	Einzel-bestimmung Gefunden in %	Doppel-bestimmung Gefunden in %
C	X	-		965 D1	3,800	(~0,1)	
H	X	0.23%		/		0,34	
N	X	-		/		/	
S	X	-		/		/	
Rb		61.5%		/			
Mo	X	23%		101 D1	5,371	23,14	
O		15.3%		/			
				/			
				/			

Besondere Hinweise: Die Probe muss absolut homogen sein! Keine Rundkolben! Keine Rührfische! — Datum: 26.08

☒ sehr luftempfindlich ☐ thermolabil ☐ giftig ☐ Aufbewahrung im Kühlschrank
☐ wenig luftempfindlich ☐ flüchtig ☐ polymer ☐ Aufbewahrung im Gefrierfach
☒ hygroskopisch ☐ explosiv ☐ flüssig ☐ stinkt
☐ lichtempfindlich ☐ elektrostatisch ☐ auf Abruf

Einwaage: ☐ offen ☒ glove-box

Bemerkungen: (wird vom Mikrolabor ausgefüllt)
☐ Gewichtszunahme ☐ Gewichtsabnahme ☐ zu geringe Einwaage
☐ .... außerhalb des Messbereichs ✗CHNS ....nur Orientierungswert

TUM Zentralinstitut für Katalyseforschung
Elementaranalyse
Ernst-Otto-Fischer Str.1
85748 Garching
Tel: +49 89 289-54127
elementaranalyse@tum.de

Datum: 27.08.2021/ B.M.
Durchgeführt von: B.D.

Öffnungszeiten Mo-Do 9-10 Uhr und 14-14.30 Uhr / Freitag 9-10 Uhr

Analysenauftrag/-bericht

Name: A. Mutschke — Telefon: 13145
Raum: 47422
Substanzbezeichnung: Cs3MoO4H — Arbeitskreis: Faessler

Bitte **alle** anwesenden Elemente mit deren ungefähren Massenprozentwerten angeben. (Summe aller Massenprozentwerte sollte 100% ergeben!)

Alle anwesenden Elemente	Zu best.	Alle ungef. %-Gehalte	Param. Zuschl.	Analysen-Nr.	Einwaage in mg	Einzel-bestimmung Gefunden in %	Doppel-bestimmung Gefunden in %
C	X	-		958 D1	3,338	(~0,2)	
H	X	0.18%		/		0,17	
N	X	-		/		/	
S	X	-		/		/	
Cs		71.2%		/			
Mo	X	17.1%		100 D1	5,186	17,14	
O		11.4%		/			
				/			
				/			

Besondere Hinweise: Die Probe muss absolut homogen sein! Keine Rundkolben! Keine Rührfische! — Datum: 26.08

☒ sehr luftempfindlich ☐ thermolabil ☐ giftig ☐ Aufbewahrung im Kühlschrank
☐ wenig luftempfindlich ☐ flüchtig ☐ polymer ☐ Aufbewahrung im Gefrierfach
☒ hygroskopisch ☐ explosiv ☐ flüssig ☐ stinkt
☐ lichtempfindlich ☐ elektrostatisch ☐ auf Abruf

Einwaage: ☐ offen ☒ glove-box

Bemerkungen: (wird vom Mikrolabor ausgefüllt)
☐ Gewichtszunahme ☐ Gewichtsabnahme ☐ zu geringe Einwaage
☐ .... außerhalb des Messbereichs ✗CHNS ....nur Orientierungswert

TUM Zentralinstitut für Katalyseforschung
Elementaranalyse
Ernst-Otto-Fischer Str.1
85748 Garching
Tel: +49 89 289-54127
elementaranalyse@tum.de

Datum: 27.08.2021/ B.M.
Durchgeführt von: B.D.

Öffnungszeiten Mo-Do 9-10 Uhr und 14-14.30 Uhr / Freitag 9-10 Uhr

Figure S28. Elemental analysis reports of the four hydridic compounds.

2.9 Optimized crystal structures as CIF (DFT-PBE0)

```
data_Rb3MoO4H
_audit_creation_method FINDSYM

_cell_length_a    7.8499197535
_cell_length_b    7.8499197535
_cell_length_c    12.4512660437
_cell_angle_alpha 90.0000000000
_cell_angle_beta  90.0000000000
_cell_angle_gamma 90.0000000000

_symmetry_space_group_name_H-M "I 4/m 2/c 2/m"
_symmetry_Int_Tables_number 140
_space_group.reference_setting '140:-I 4 2c'
_space_group.transform_Pp_abc a,b,c;0,0,0

loop_
_space_group_symop_id
_space_group_symop_operation_xyz
1 x,y,z
2 x,-y,-z+1/2
3 -x,y,-z+1/2
4 -x,-y,z
5 -y,-x,-z+1/2
6 -y,x,z
7 y,-x,z
8 y,x,-z+1/2
9 -x,-y,-z
10 -x,y,z+1/2
11 x,-y,z+1/2
12 x,y,-z
13 y,x,z+1/2
14 y,-x,-z
15 -y,x,-z
16 -y,-x,z+1/2
17 x+1/2,y+1/2,z+1/2
18 x+1/2,-y+1/2,-z
19 -x+1/2,y+1/2,-z
20 -x+1/2,-y+1/2,z+1/2
21 -y+1/2,-x+1/2,-z
22 -y+1/2,x+1/2,z+1/2
23 y+1/2,-x+1/2,z+1/2
24 y+1/2,x+1/2,-z
25 -x+1/2,-y+1/2,-z+1/2
26 -x+1/2,y+1/2,z
27 x+1/2,-y+1/2,z
28 x+1/2,y+1/2,-z+1/2
29 y+1/2,x+1/2,z
30 y+1/2,-x+1/2,-z+1/2
31 -y+1/2,x+1/2,-z+1/2
32 -y+1/2,-x+1/2,z

loop_
_atom_site_label
_atom_site_type_symbol
_atom_site_symmetry_multiplicity
_atom_site_Wyckoff_label
_atom_site_fract_x
_atom_site_fract_y
_atom_site_fract_z
_atom_site_occupancy
Mo1 Mo   4 b 0.00000 0.50000 0.25000 1.00000
Rb1 Rb   8 h 0.18599 0.68599 0.00000 1.00000
Rb2 Rb   4 a 0.00000 0.00000 0.25000 1.00000
O1  O   16 l 0.62870 0.12870 0.16569 1.00000
H1  H    4 c 0.00000 0.00000 0.00000 1.00000

# end of cif




data_Cs3MoO4H
_audit_creation_method FINDSYM

_cell_length_a    8.2190168880
_cell_length_b    8.2190168880
```

```
_cell_length_c    12.9251378714
_cell_angle_alpha 90.0000000000
_cell_angle_beta  90.0000000000
_cell_angle_gamma 90.0000000000

_symmetry_space_group_name_H-M "I 4/m 2/c 2/m"
_symmetry_Int_Tables_number 140
_space_group.reference_setting '140:-I 4 2c'
_space_group.transform_Pp_abc a,b,c;0,0,0

loop_
_space_group_symop_id
_space_group_symop_operation_xyz
1 x,y,z
2 x,-y,-z+1/2
3 -x,y,-z+1/2
4 -x,-y,z
5 -y,-x,-z+1/2
6 -y,x,z
7 y,-x,z
8 y,x,-z+1/2
9 -x,-y,-z
10 -x,y,z+1/2
11 x,-y,z+1/2
12 x,y,-z
13 y,x,z+1/2
14 y,-x,-z
15 -y,x,-z
16 -y,-x,z+1/2
17 x+1/2,y+1/2,z+1/2
18 x+1/2,-y+1/2,-z
19 -x+1/2,y+1/2,-z
20 -x+1/2,-y+1/2,z+1/2
21 -y+1/2,-x+1/2,-z
22 -y+1/2,x+1/2,z+1/2
23 y+1/2,-x+1/2,z+1/2
24 y+1/2,x+1/2,-z
25 -x+1/2,-y+1/2,-z+1/2
26 -x+1/2,y+1/2,z
27 x+1/2,-y+1/2,z
28 x+1/2,y+1/2,-z+1/2
29 y+1/2,x+1/2,z
30 y+1/2,-x+1/2,-z+1/2
31 -y+1/2,x+1/2,-z+1/2
32 -y+1/2,-x+1/2,z

loop_
_atom_site_label
_atom_site_type_symbol
_atom_site_symmetry_multiplicity
_atom_site_Wyckoff_label
_atom_site_fract_x
_atom_site_fract_y
_atom_site_fract_z
_atom_site_occupancy
Mo1 Mo   4 b 0.00000 0.50000 0.25000 1.00000
Cs1 Cs   8 h 0.18530 0.68530 0.00000 1.00000
Cs2 Cs   4 a 0.00000 0.00000 0.25000 1.00000
O1  O   16 l 0.62336 0.12336 0.16911 1.00000
H1  H    4 c 0.00000 0.00000 0.00000 1.00000

# end of cif



data_Cs3WO4H
_audit_creation_method FINDSYM

_cell_length_a    8.2391299711
_cell_length_b    8.2391299711
_cell_length_c    12.8404999808
_cell_angle_alpha 90.0000000000
_cell_angle_beta  90.0000000000
_cell_angle_gamma 90.0000000000

_symmetry_space_group_name_H-M "I 4/m 2/c 2/m"
_symmetry_Int_Tables_number 140
```

```
_space_group.reference_setting '140:-I 4 2c'
_space_group.transform_Pp_abc a,b,c;0,0,0

loop_
_space_group_symop_id
_space_group_symop_operation_xyz
1 x,y,z
2 x,-y,-z+1/2
3 -x,y,-z+1/2
4 -x,-y,z
5 -y,-x,-z+1/2
6 -y,x,z
7 y,-x,z
8 y,x,-z+1/2
9 -x,-y,-z
10 -x,y,z+1/2
11 x,-y,z+1/2
12 x,y,-z
13 y,x,z+1/2
14 y,-x,-z
15 -y,x,-z
16 -y,-x,z+1/2
17 x+1/2,y+1/2,z+1/2
18 x+1/2,-y+1/2,-z
19 -x+1/2,y+1/2,-z
20 -x+1/2,-y+1/2,z+1/2
21 -y+1/2,-x+1/2,-z
22 -y+1/2,x+1/2,z+1/2
23 y+1/2,-x+1/2,z+1/2
24 y+1/2,x+1/2,-z
25 -x+1/2,-y+1/2,-z+1/2
26 -x+1/2,y+1/2,z
27 x+1/2,-y+1/2,z
28 x+1/2,y+1/2,-z+1/2
29 y+1/2,x+1/2,z
30 y+1/2,-x+1/2,-z+1/2
31 -y+1/2,x+1/2,-z+1/2
32 -y+1/2,-x+1/2,z

loop_
_atom_site_label
_atom_site_type_symbol
_atom_site_symmetry_multiplicity
_atom_site_Wyckoff_label
_atom_site_fract_x
_atom_site_fract_y
_atom_site_fract_z
_atom_site_occupancy
W1  W    4 b 0.00000 0.50000 0.25000 1.00000
Cs1 Cs   8 h 0.18892 0.68892 0.00000 1.00000
Cs2 Cs   4 a 0.00000 0.00000 0.25000 1.00000
O1  O   16 l 0.62510 0.12510 0.16680 1.00000
H1  H    4 c 0.00000 0.00000 0.00000 1.00000

# end of cif




data_Rb3WO4H
_audit_creation_method FINDSYM

_cell_length_a    11.9743502500
_cell_length_b    11.4712919800
_cell_length_c    11.5041418900
_cell_angle_alpha 90.0000000000
_cell_angle_beta  90.0000000000
_cell_angle_gamma 90.0000000000

_symmetry_space_group_name_H-M "P 21/b 21/c 21/a"
_symmetry_Int_Tables_number 61
_space_group.reference_setting '061:-P 2ac 2ab'
_space_group.transform_Pp_abc a,b,c;0,0,0

loop_
_space_group_symop_id
_space_group_symop_operation_xyz
1 x,y,z
```

```
2 x+1/2,-y+1/2,-z
3 -x,y+1/2,-z+1/2
4 -x+1/2,-y,z+1/2
5 -x,-y,-z
6 -x+1/2,y+1/2,z
7 x,-y+1/2,z+1/2
8 x+1/2,y,-z+1/2

loop_
_atom_site_label
_atom_site_type_symbol
_atom_site_symmetry_multiplicity
_atom_site_Wyckoff_label
_atom_site_fract_x
_atom_site_fract_y
_atom_site_fract_z
_atom_site_occupancy
W1  W    8 c 0.74991 0.00791  -0.01757 1.00000
Rb1 Rb   8 c 0.74671 0.22901  0.72928  1.00000
Rb2 Rb   8 c 0.00202 0.78349  -0.00761 1.00000
Rb3 Rb   8 c 0.00245 -0.00693 0.71810  1.00000
O1  O    8 c 0.86839 -0.02153 0.07208  1.00000
O2  O    8 c 0.78097 -0.02617 0.83424  1.00000
O3  O    8 c 0.71730 0.15992  -0.00939 1.00000
O4  O    8 c 0.63217 -0.07532 0.02845  1.00000
H1  H    8 c 0.50535 0.75489  0.25699  1.00000

# end of cif
```

3 Literature

[1] J. Rodríguez-Carvajal, *Physica B* **1993**, *192*, 55.

[2] J. Tauc, R. Grigorovici, A. Vancu, *Phys. Status Solidi* **1966**, *15*, 627.

[3] R. Dovesi, A. Erba, R. Orlando, C. M. Zicovich-Wilson, B. Civalleri, L. Maschio, M. Rérat, S. Casassa, J. Baima, S. Salustro et al., *Wiley Interdiscip. Rev.-Comput. Mol. Sci.* **2018**, *8*.

[4] Perdew, Burke, Ernzerhof, *Phys. Rev. Lett.* **1996**, *77*, 3865.

[5] C. Adamo, V. Barone, *J. Chem. Phys.* **1999**, *110*, 6158.

[6] F. Weigend, R. Ahlrichs, *Phys. Chem. Chem. Phys.* **2005**, *7*, 3297.

[7] a) R. E. Stene, B. Scheibe, A. J. Karttunen, W. Petry, F. Kraus, *Eur. J. Inorg. Chem.* **2019**, *2019*, 3672; b) R. E. Stene, B. Scheibe, A. J. Karttunen, W. Petry, F. Kraus, *Eur. J. Inorg. Chem.* **2020**, *2020*, 2260; c) A. J. Karttunen, T. Tynell, M. Karppinen, *J. Phys. Chem. C* **2015**, *119*, 13105.

[8] H. J. Monkhorst, J. D. Pack, *Phys. Rev. B* **1976**, *13*, 5188.

[9] a) F. Pascale, C. M. Zicovich-Wilson, F. López Gejo, B. Civalleri, R. Orlando, R. Dovesi, *J. Comput. Chem.* **2004**, *25*, 888; b) C. M. Zicovich-Wilson, F. Pascale, C. Roetti, V. R. Saunders, R. Orlando, R. Dovesi, *J. Comput. Chem.* **2004**, *25*, 1873; c) L. Maschio, B. Kirtman, M. Rérat, R. Orlando, R. Dovesi, *J. Chem. Phys.* **2013**, *139*, 164101.

[10] a) S. J. Clark, M. D. Segall, C. J. Pickard, P. J. Hasnip, M. I. J. Probert, K. Refson, M. C. Payne, *Z. Kristallogr. – Cryst. Mater.* **2005**, *220*, 567; b) C. J. Pickard, F. Mauri, *Phys. Rev. B* **2001**, *63*; c) J. R. Yates, C. J. Pickard, F. Mauri, *Phys. Rev. B* **2007**, *76*.

[11] Vanderbilt, *Phys. Rev. B* **1990**, *41*, 7892.

[12] R. D. Shannon, *Acta Cryst. A* **1976**, *32*, 751.

[13] P. F. Lang, B. C. Smith, *Dalton Trans.* **2010**, *39*, 7786.

[14] K. Momma, F. Izumi, *J. Appl. Crystallogr.* **2011**, *44*, 1272.

[15] Y. Hinuma, G. Pizzi, Y. Kumagai, F. Oba, I. Tanaka, *Band structure diagram paths based on crystallography*, **2016**.

[16] A. Togo, I. Tanaka **2018**, arXiv:1808.01590v1.

[17] Y. Hinuma, G. Pizzi, Y. Kumagai, F. Oba, I. Tanaka, *Comput. Mater. Sci.* **2017**, *128*, 140.
